# Quantitative SPECT/CT imaging of lead-212: a phantom study

**DOI:** 10.1186/s40658-022-00481-z

**Published:** 2022-08-04

**Authors:** Monika Kvassheim, Mona-Elisabeth R. Revheim, Caroline Stokke

**Affiliations:** 1 Department of Physics and Computational Radiology, Division of Radiology and Nuclear Medicine, Oslo University Hospital, Oslo, Norway; 2 Faculty of Medicine, University of Oslo, Oslo, Norway; 3 Department of Nuclear Medicine, Division of Radiology and Nuclear Medicine, Oslo University Hospital, Oslo, Norway; 4 Department of Physics, University of Oslo, Oslo, Norway

**Keywords:** Lead-212, Alpha particle, Therapy, Imaging, SPECT, Phantom, Quantitative

## Abstract

**Background:**

Lead-212 (^212^Pb) is a promising radionuclide for targeted therapy, as it decays to α-particle emitter bismuth-212 (^212^Bi) via β-particle emission. This extends the problematic short half-life of ^212^Bi. In preparation for upcoming clinical trials with ^212^Pb, the feasibility of quantitative single photon-emission computed tomography/computed tomography (SPECT/CT) imaging of ^212^Pb was studied, with the purpose to explore the possibility of individualised patient dosimetric estimation.

**Results:**

Both acquisition parameters (combining two different energy windows and two different collimators) and iterative reconstruction parameters (varying the iterations x subsets between 10 × 1, 15 × 1, 30 × 1, 30 × 2, 30 × 3, 30 × 4, and 30 × 30) were investigated to evaluate visual quality and quantitative uncertainties based on phantom images. Calibration factors were determined using a homogeneous phantom and were stable when the total activity imaged exceeded 1 MBq for all the imaging protocols studied, but they increased sharply as the activity decayed below 1 MBq. Both a 20% window centred on 239 keV and a 40% window on 79 keV, with dual scatter windows of 5% and 20%, respectively, could be used. Visual quality at the lowest activity concentrations was improved with the High Energy collimator and the 79 keV energy window. Fractional uncertainty in the activity quantitation, including uncertainties from calibration factors and small volume effects, in spheres of 2.6 ml in the NEMA phantom was 16–21% for all protocols with the 30 × 4 filtered reconstruction except the High Energy collimator with the 239 keV energy window. Quantitative analysis was possible both with and without filters, but the visual quality of the images improved with a filter.

**Conclusions:**

Only minor differences were observed between the imaging protocols which were all determined suitable for quantitative imaging of ^212^Pb. As uncertainties generally decreased with increasing iterative updates in the reconstruction and recovery curves did not converge with few iterations, a high number of reconstruction updates are recommended for quantitative imaging.

**Supplementary Information:**

The online version contains supplementary material available at 10.1186/s40658-022-00481-z.

## Background

Targeted radioligand therapy consists of selectively delivering radiation to cancer cells using targeting agents and has been proven a successful treatment for several cancers. Currently, beta (β)-particle emitters are the main radionuclides used; however, using alpha (α)-particle emitters in targeted alpha therapy (TAT) presents an appealing alternative. Compared to β-particles, α-particles have higher energies, shorter path lengths, and much higher probabilities of DNA double-strand breaks when interacting with cell nuclei [1, 2]. These are all properties desirable for targeting small tumours in metastasised cancer as they allow for high absorbed doses to cancerous tissue while avoiding toxicity in surrounding tissue. The cytotoxicity of α-particles is approximately 500 times higher than that of β-particles and a high level of accuracy in delivering the α-radiation is essential to avoid damage to non-targeted tissues [3]. This accuracy may be assessed with imaging, but imaging of α-particle emitters is difficult due to few photons of appropriate energies being emitted at the relevant activity levels.

Several α-particle emitters have already been studied in patients and many of them have been imaged. Radium-223 (^223^Ra) is an α-particle emitter currently used in the form of ^223^Ra-dichloride to treat bone metastases, and it is the most studied α-particle emitter in radiotherapy [4–7]. Quantitative planar scintigraphy and single photon-emission computed tomography (SPECT) imaging have been investigated for ^223^Ra and were found to be potentially useful tools for dosimetry [4]. α-particle therapies have been proposed for a variety of cancers with different targeting agents, and an overview of TATs on the market or in development was provided recently by Sgouros et al. [8]. Imaging of several of the α-particle emitting radionuclides has been shown to be feasible; for instance, thorium-227 (^227^Th) and actinium-225 (^225^Ac) can be imaged with planar scintigraphy [5, 6, 9–13] and SPECT [11–14]. Planar scintigraphy and SPECT scans of the liver and brain have been performed with bismuth-213 (^213^Bi) [14–16], while terbium-149 (^149^Tb) can be imaged with positron emission tomography (PET) [17]. ^212^Pb functions as an in vivo α-particle emitter generator as it decays to bismuth-212 (^212^Bi) with a half-life of 10.64 h. As ^212^Pb decays to ^212^Bi, imageable gammas of 238.6 keV, with an intensity of 43.3%, and X-rays (75–91 keV), with a total intensity of 36%, are emitted [18]. Planar scintigraphy images of ^212^Pb were obtained by Meredith et al. in a clinical study [19] and by Kasten et al. in a preclinical study [20]. Surrogate radiopharmaceuticals with more appropriate photon emissions are sometimes used [21], and lead-203 (^203^Pb) has been investigated as a surrogate isotope for ^212^Pb [22, 23]. As there are photon emissions in the ^212^Pb decay chain that are imageable, we here investigate the possibilities for direct quantitative SPECT imaging of ^212^Pb.

There are several good targets for targeted radioligand therapy using ^212^Pb. Prostate-specific membrane antigen (PSMA) TATs could be an improvement on current treatments available for patients with castration-resistant metastatic prostate cancer, and ^212^Pb is an advantageous choice of radionuclide due to the industrial-scale production methods available [24]. Encouraging preclinical results with PSMA-targeting ^212^Pb-NG001 show lower kidney uptake than ^212^Pb-labelled PSMA-617 in addition to good tumour uptake and inhibited tumour growth [25, 26], and a clinical trial is being planned. The current imaging study has been conducted using a Siemens Symbia Intevo Bold to prepare for imaging and dosimetry of patients injected with ^212^Pb-targeted radiotherapy. Collimators, energy windows, and reconstruction parameters have been compared using various phantoms. The aim of this study was to determine the feasibility of and to find the best protocol for quantitative SPECT/computed tomography (CT) imaging of ^212^Pb.

## Materials and methods

### Decay characteristics

**Fig. 1:**
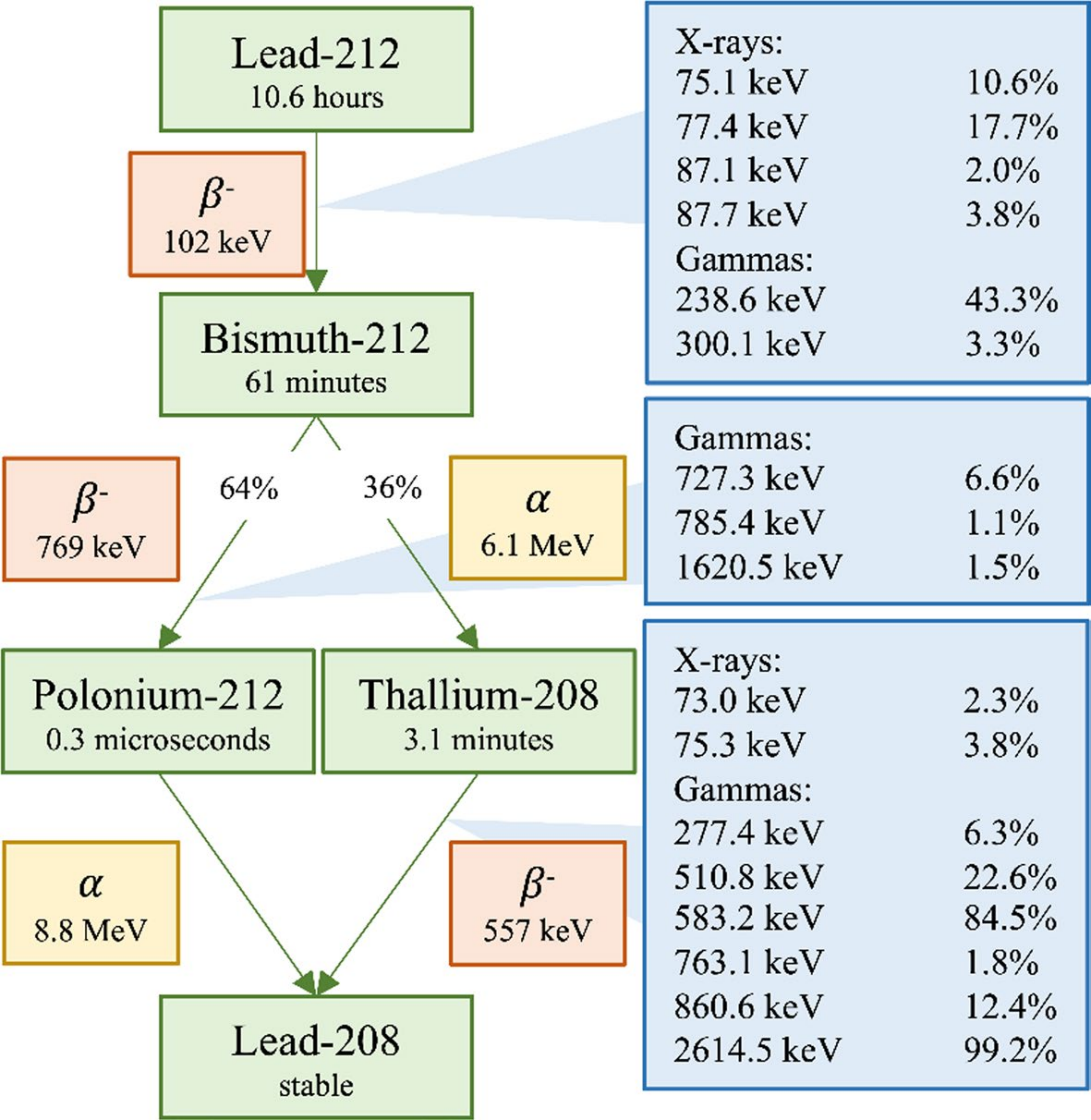
Decay scheme of ^212^Pb. The mean alpha and beta energies are included. The relevant photons emitted and their energies are added to the transition. Photons with emission probabilities smaller than 1% per ^212^Pb decay or with energies below 70 keV are not included. The data are taken from ICRP 107 [18]

^212^Pb decays via β-particle emission to α-particle emitter bismuth-212 (^212^Bi) with a half-life of 10.64 h. In the same transition, most of the photons in the decay chain are emitted, most notably one of 238.6 keV with an intensity of 43.3%. X-rays (75–91 keV) with a total intensity of 36% are also emitted during this transition. A high-energy photon emission of 2614.5 keV is emitted by daughter thallium-208 (^208^Tl) with 36% intensity per ^212^Pb decay and creates scatter which may also contribute to the SPECT energy spectrum [18]. The decay scheme and emitted photon, alpha, and beta energies are presented in Fig. 1.

### Imaging protocol

**Fig. 2:**
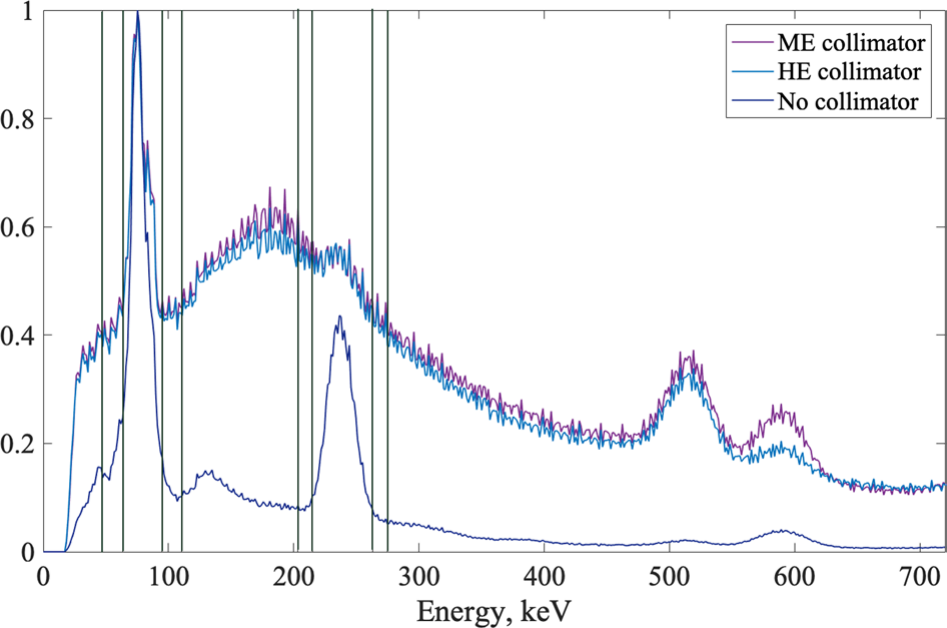
Energy spectra acquired on the SPECT scanner with a ^212^Pb source. The two investigated energy windows and their adjacent scatter windows are also illustrated. The spectrum with the ME collimator is shown in purple, the spectrum with the HE collimator is shown in light blue, and the spectrum acquired without a collimator is shown in dark blue

Four 30-min imaging protocols were examined on a Siemens Symbia Intevo Bold SPECT/CT with a 3/8″ crystal. Two collimators, High Energy (HE) and Medium Energy (ME), and two energy windows, 40% at 79 keV and 20% at 239 keV, were combined in the protocols. Dual scatter windows of 20% for the 79 keV peak centred on 55 keV and 103 keV and 5% for the 239 keV peak centred on 209 keV and 268 keV were used for scatter correction. The energy and scatter windows were chosen based on the energy spectra shown in Fig. 2. The SPECT images were acquired with body contouring orbits, a 256 × 256 matrix, and 60 views with acquisition during steps. The images were reconstructed with Flash-3D, a commercial OSEM reconstruction algorithm available on the Siemens SPECT, which includes depth-dependent 3D resolution recovery with Gaussian point spread function correction [27]. Reconstruction updates are here used for number of iterations multiplied with number of subsets, and images were reconstructed with seven different numbers of updates (iterations x subsets): 10 × 1, 15 × 1, 30 × 1, 30 × 2, 30 × 3, 30 × 4, and 30 × 30. Images were acquired separately for the two energy windows, and attenuation correction for the central energy based on the CT images was performed. Both images with a 12-mm Gaussian filter and without filters were analysed. These imaging protocols were used for all the experiments.

### Calibration factors

Calibration factors (CFs) were determined by imaging a 6283 ml uniform cylindrical phantom filled with 0.5–8.5 MBq ^212^Pb eleven times. In Slicer (v4, http://www.slicer.org) [28], a cylindrical volume of interest (VOI) of approximately 4000 ml was placed well within the boundaries of the phantom to avoid contributions from edge effects. The mean counts per voxel was extracted and divided by the volume of a voxel to give mean counts per ml, $\overline{C}_{{{\text{mean}}}}$, and the CF was calculated with

1 $$CF = \frac{{\overline{C}_{{{\text{mean}}}} }}{{t \times \overline{A}}} ,$$

where *t* is the scan duration and $\overline{A}$ is the activity per ml.

### Recovery coefficients and contrast

The National Electrical Manufacturers Association (NEMA) International Electrotechnical Commission (IEC) PET Body Phantom was used to find mean and maximum recovery coefficients, RC_mean_ and RC_max_, and to determine the contrast achieved with the different imaging protocols. The sphere volumes were 26.52, 11.49, 5.57, 2.57, 1.15, and 0.52 ml. The phantom was filled twice, with ^212^Pb in the spheres and non-radioactive water in the background. For each imaging protocol, the phantom was imaged with eight different activity concentrations in the spheres, between 17.2 kBq/ml and 104.2 kBq/ml, that is a total of 0.8–5.0 MBq. The maximum and mean counts per ml, $\overline{C}_{{{\text{max}}}}$ and $\overline{C}_{{{\text{mean}}}}$, in each sphere were obtained by placing spherical VOIs of the physical sphere volume using the CT images in Slicer, extracting maximum and mean counts per voxel, and dividing by voxel size. The mean counts per ml in the background, $\overline{C}_{{{\text{background}}}}$, resulting from scatter and spill-out, were calculated by placing a cylinder VOI of radius 20 mm and height 18 mm in the volume between the spheres.

The data points for the recovery coefficient fit were calculated using

2 $$RC = \frac{{\left( {\overline{C} - \overline{C}_{{{\text{background}}}} } \right)}}{{CF \times t \times \overline{A}_{{\text{true, sph}}} }},$$

where *t* is the scan duration and $\overline{A}_{{\text{true, sph}}}$ is the known, physical activity concentration in the sphere. RC is the recovery coefficient which is RC_max_ if $\overline{C}_{{{\text{max}}}}$ is used for $\overline{C}$ and RC_mean_ if $\overline{C}_{{{\text{mean}}}}$ is used for $\overline{C}$. The uncertainties on the activity quantitation of the volumes were propagated from the standard deviations (SDs) of $\overline{C}_{{\text{mean, max}}}$ and CFs. Fractional uncertainties were taken as this uncertainty divided by the average of the eight RC_mean, max_ for each volume. The recovery coefficient data points were fitted to the function *RC*_fit_ with

3 $$RC_{{{\text{fit}}}} = 1 - \frac{1}{{1 + \frac{v}{{b_{1} }}^{{b_{2} }} }},$$

where *v* is volume and *b*_1_ and *b*_2_ are fitting parameters [29]. The fit parameters and associated standard errors (SE) were calculated using MATLAB (R2017a, MathWorks). The eight RC_mean_ data points for each volume were included in the fit, not the average used for fractional uncertainties.

The contrast was calculated as

4 $${\text{Contrast}} = \frac{{\overline{C}_{{{\text{mean}}}} - \overline{C}_{{{\text{background}}}} }}{{\overline{C}_{{{\text{mean}}}} }},$$

where the symbols are the same as defined above. The errors on contrast were taken as the SDs of measurements at different activity levels.

## Results

**Fig. 3:**
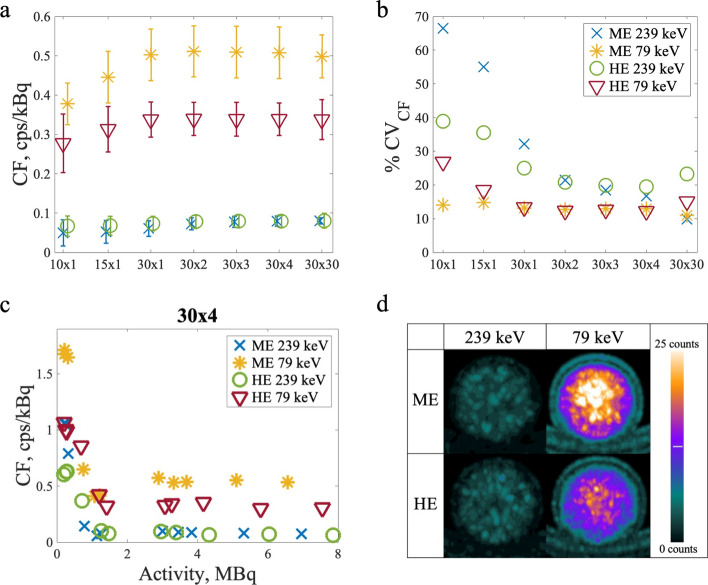
Results for the CFs for the reconstructions with a filter applied with ^212^Pb. In panel **a**, the CFs are plotted for different reconstructions with error bars showing the SDs of the CFs calculated at activities above 1 MBq. In panel **b**, the plot shows CVs of the CFs plotted for different reconstructions. The corresponding results for images without a filter can be found in the supplementary material. In panel **c**, the data points from which the CFs were calculated for the 30 × 4 reconstruction are plotted against activity for all four image protocols with a filter applied. The sharp increase in CFs as activity decays below 1 MBq can be seen in the plot. In panel **d**, high activity maximum intensity projection images of the homogeneous phantom are presented for each of the imaging protocols with a 12-mm filter and the 30 × 4 reconstruction. The scale is set the same for all the images, to illustrate the different count rates, with the white set to 25 counts and the black being 0 counts

The calibration factors calculated for the four imaging protocols with a filter are presented in Fig. 3. Corresponding results for the unfiltered images can be found in Additional file 1. The CFs stayed approximately constant for all protocols when more than 1 MBq (0.16 kBq/ml) ^212^Pb was imaged, but increased sharply below 1 MBq. Due to this, only data points obtained with more activity than 1 MBq were used when calculating the average CFs. The count rate was highest with the ME collimator and the 79 keV window, giving a CF_ME,79 keV_ = 0.51 ± 0.07 cps/kBq when taking the mean and SD of all the values obtained with activities higher than 1 MBq. The count rate decreased when the HE collimator was used with the same energy window, giving CF_HE,79 keV_ = 0.34 ± 0.04 cps/kBq. As can be seen from Fig. 3a, the 239 keV window gave the lowest count rate and little difference between the collimators, giving CF_ME,239 keV_ = 0.08 ± 0.01 cps/kBq and CF_HE,239 keV_ = 0.08 ± 0.02 cps/kBq. The CFs for the reconstructions without a filter and the other numbers of updates are given in Additional file 1 and show very similar values. As seen in Fig. 3a, there was little variation in CFs between reconstructions except a dip for the 10 × 1 and 15 × 1 reconstructions. The dip was most significant for image protocols using the 79 keV peak and largest for the ME collimator. The ME 79 keV was the image protocol which most consistently had the lowest coefficients of variation (CVs) on the CFs (Fig. 3b). This imaging protocol also had approximately constant CVs with reconstruction updates, as they stayed between 11 and 15% for all reconstructions when a filter was applied. The CVs for HE 79 keV only deviated from the ME 79 keV CVs level for 10 × 1 and 15 × 1, with 27% and 18%, respectively. The 239 keV window caused larger CVs, but while for the HE collimator the CVs varied between 20 and 39%, the ME collimator gave a 66% CV for 10 × 1 which steadily decreased with increasing iteration updates to 10% for 30 × 30. These trends were similar for the reconstructions without a filter.

**Fig. 4:**
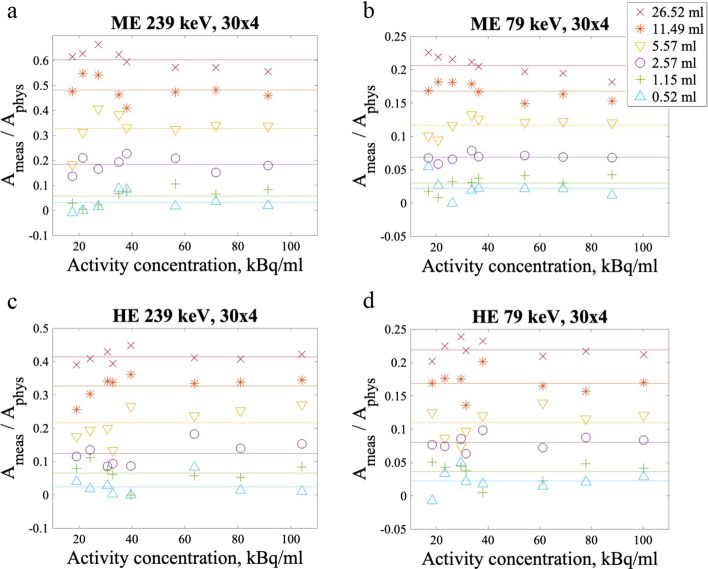
Activity in each sphere measured from the mean counts in the images divided by the known, physical activity in each sphere plotted against activity concentration. The figure shows the results obtained with the filtered 30 × 4 reconstruction, with ME 239 keV in panel **a**, ME 79 keV in panel **b**, HE 239 keV in panel **c**, and HE 79 keV in panel **d**. Equivalent figures for the other reconstructions with and without filters applied can be found in the supplementary material. The mean activity ratio for the sphere volume is shown as a line. Of note, the x-axes are the same, but the values on the y-axes differ

Using the calculated CFs, the measured mean counts in the six spheres of the NEMA phantom were converted to activity concentrations. The measured activity concentrations were divided by the known physical activity concentrations for the six spheres at different time points and plotted against activity concentration. The results for the 30 × 4 reconstruction with a filter applied are presented in Fig. 4, and the equivalent plots for the other reconstructions are included in Additional file 1.

**Fig. 5:**
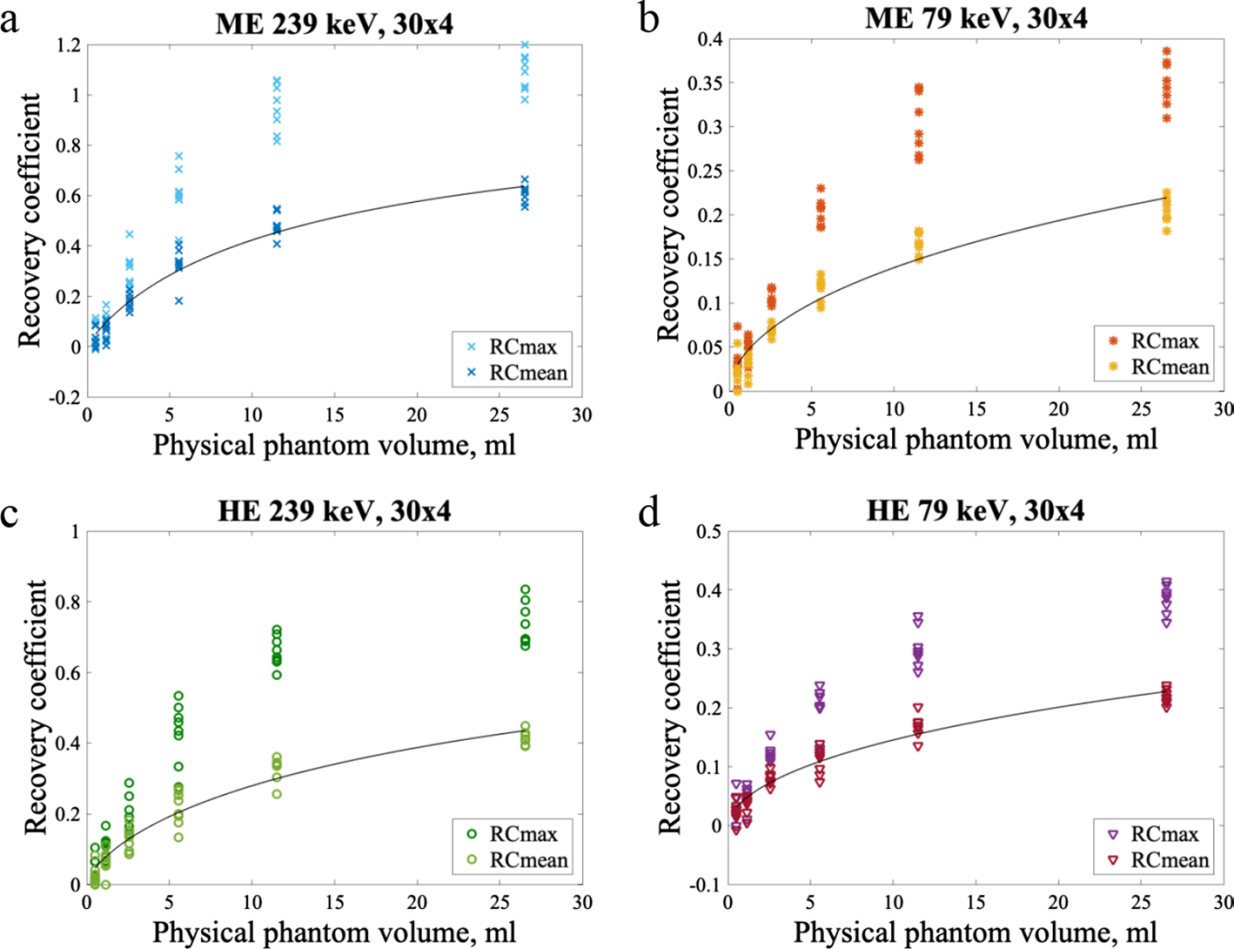
RCs and curve fits for the four image protocols with the 30 × 4 reconstruction and a 12-mm Gaussian filter. The equivalent figures for the other reconstructions can be found in the supplementary material. ME 239 keV is in panel **a**, ME 79 keV is in panel **b**, HE 239 keV is in panel **c**, and HE 79 keV is in panel **d**. Both RC_max_ and RC_mean_ data points are plotted, in addition to a curve fit to RC_mean_

Recovery coefficient (RC) curves are shown for the 30 × 4 reconstruction with a filter applied in Fig. 5. Both RC_mean_ and RC_max_ values are plotted, and the curves fit to RC_mean_ are also shown. The intensity spread, used here for all effects that in an image contribute to spread of intensity around a source such as spill-out and partial volume effects, appeared to be more significant for the 79 keV window. The curve fit parameters and the associated SE for the 30 × 4 reconstruction with a filter applied are presented in Table 1. The fractional SE on the curve fit parameters were smaller than or equal to 16% for all protocols with the 30 × 4 reconstruction. The fractional SE were smaller for the 239 keV window than the 79 keV window for both collimators. Corresponding figures and tables can be found for the other reconstructions, with and without a filter applied, in the supplementary material. The fractional SE were similar for the other reconstructions, except for the 30 × 30 reconstruction without a filter applied which had fractional SE exceeding 50% on *b*_*1*_ with the 79 keV window. The fitted functions were different with fewer reconstruction updates, as the steepness of the curve at small volumes increased with the number of updates. With few reconstruction updates, the recovery curves did not converge. Without a filter applied, there was more variation in RC_max_ values, especially with high numbers of updates.

**Table 1:** Fitting parameters for the RC curve for the 30 × 4 reconstruction with a 12-mm Gaussian filter

RC_mean_, 30 × 4	Parameter	Value	Standard error	Fractional standard error (%)
ME 239 keV	*b*_1_	14.14 ml	0.7270 ml	5
	*b*_2_	0.8903	0.0449	5
ME 79 keV	*b*_1_	256.9 ml	40.56 ml	16
	*b*_2_	0.559	0.0278	5
HE 239 keV	*b*_1_	38.29 ml	3.153 ml	8
	*b*_2_	0.7067	0.0376	5
HE 79 keV	*b*_1_	231.5 ml	35.03 ml	15
	*b*_2_	0.5639	0.0278	5

**Fig. 6:**
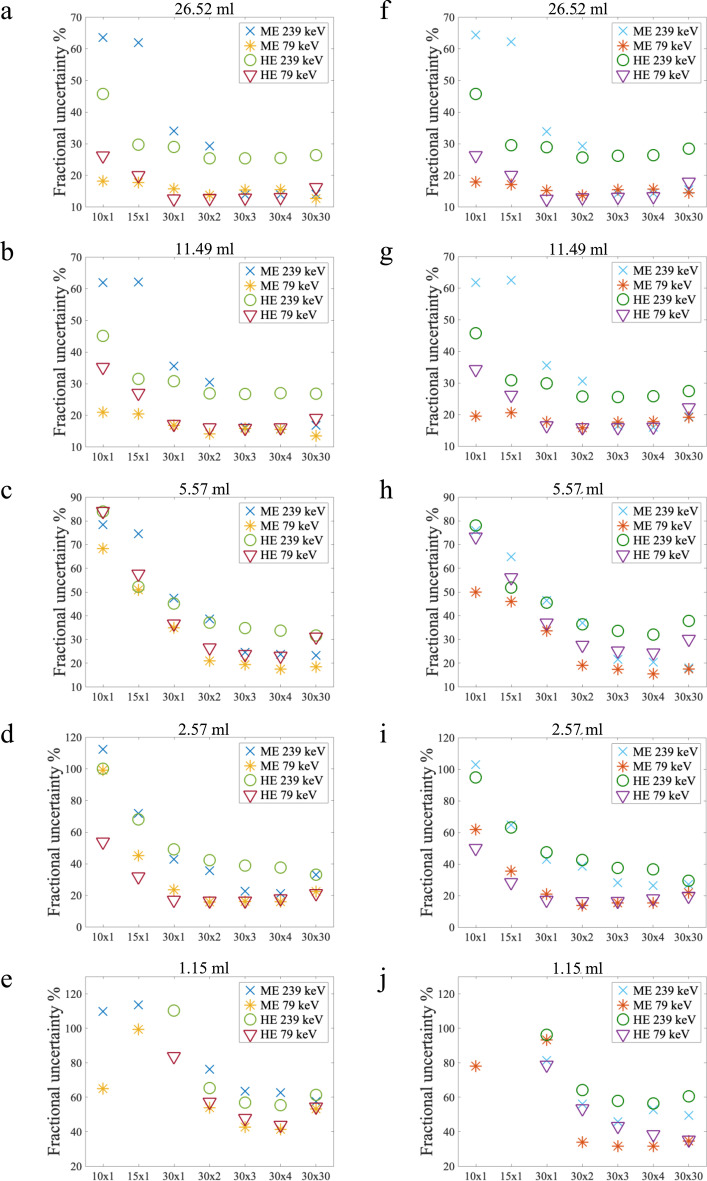
Fractional uncertainties for activity quantitation in spheres plotted against reconstruction updates for different sphere volumes. Results from using the mean counts are on the left (**a**–**e**), and results from using maximum counts are on the right (**f**–**j**). The smallest sphere (0.52 ml) is not included as all the uncertainties exceeded 60%

The fractional uncertainties on the activity quantitation in the spheres were taken as the combined uncertainties from the CFs and measured counts in the spheres, divided by the average RC for the volume, and they are plotted against reconstruction updates for different volumes in Fig. 6. The fractional uncertainties were similar for ME 239 keV, ME 79 keV, and HE 79 keV with the three reconstructions with most iteration updates. The HE 239 keV protocol consistently gave larger fractional uncertainties. With fewer reconstruction updates than 30 × 3, the fractional uncertainties of both HE 239 keV and ME 239 keV were markedly larger than those of the 79 keV window. With the 30 × 4 reconstruction, the fractional uncertainty on activity quantitation when using maximum counts for the 2.57 ml sphere was 15% for the ME 79 keV protocol and 18% for the HE 79 keV protocol with the 30 × 4 reconstruction. For activity quantitation with mean counts, with the same sphere and reconstruction, the fractional uncertainty was 16% for ME 79 keV and 18% for HE 79 keV. 2.57 ml was the smallest sphere for which activity quantitation with fractional uncertainties below 20% was possible with ^212^Pb. The corresponding plots for reconstructions without a filter applied are included in Additional file 1. The trends and values were similar with and without a filter, but without a filter the fractional uncertainties for activity quantitation using maximum counts with the 30 × 30 reconstruction increased for all volumes.

**Fig. 7:**
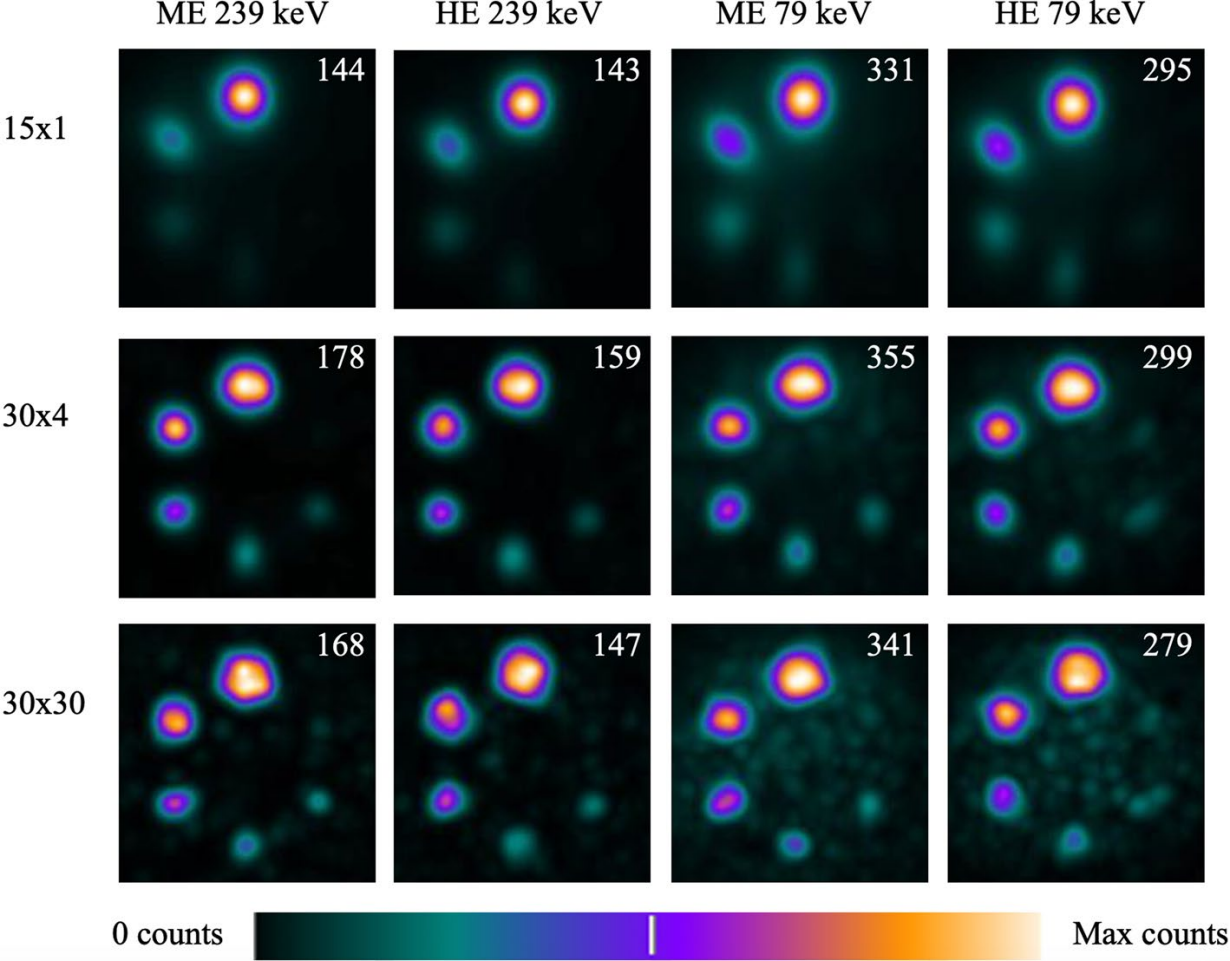
Some of the filtered reconstructed images of the NEMA phantom filled with 88–100 kBq/ml ^212^Pb for all the imaging protocols, but the maximum counts illustrated as white differs and is given in the top right corner of each image. Reconstructions 15 × 1, 30 × 4, and 30 × 30 were chosen as examples to illustrate the change with reconstruction updates

Some of the filtered reconstructed images of the NEMA phantom filled with the highest imaged concentration of ^212^Pb, 88–100 kBq/ml, are shown in Fig. 7. The smallest clearly visible sphere was the 13-mm-diameter sphere for all protocols, except the 30 × 30 reconstruction with ME 239 keV which also clearly showed the 10-mm sphere. One could make out the 10-mm sphere with the 79 keV window with the 30 × 4 reconstruction as well, but it was not clearly defined above the noise. The images appear to converge with fewer reconstruction updates for the 79 keV energy window.

**Fig. 8:**
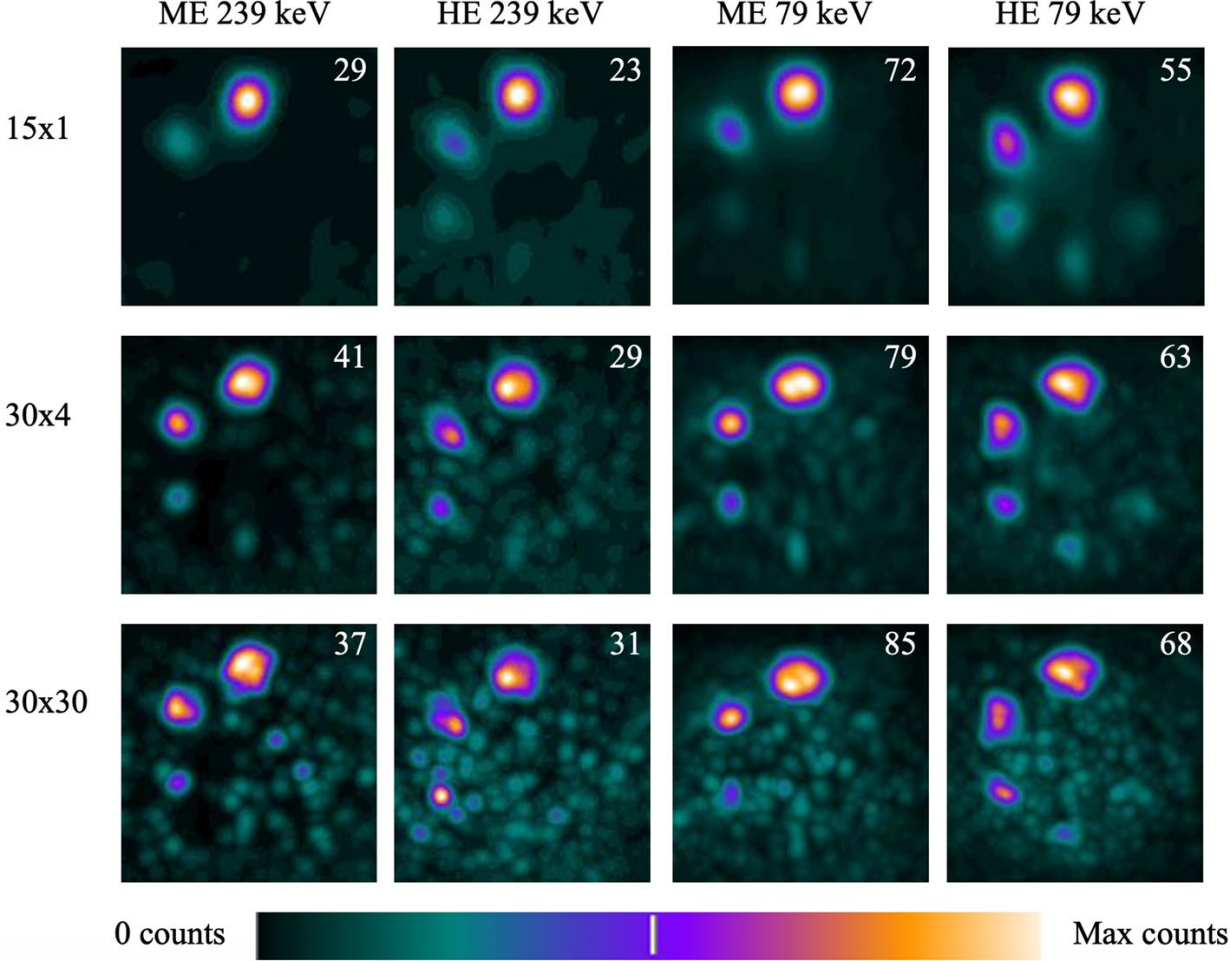
Selected filtered reconstructed images of the NEMA phantom filled with 17–19 kBq/ml ^212^Pb shown as maximum intensity projections. The scale bar is the same for all images, but the maximum counts illustrated in white differs and is given in the top right corner of each image

Selected filtered reconstructed images of the NEMA phantom with a low concentration of ^212^Pb, 17–19 kBq/ml, are shown in Fig. 8. The noise level increased compared to the high activity images for all protocols. The 30 × 30 reconstruction contained the most noise for all protocols, and it was difficult to distinguish true activity regions from noise. For the 30 × 4 reconstruction, there was also significant noise, but for the HE 79 keV images five of the spheres could be distinguished. The ME 239 keV images had the least noise and four spheres could be visualised. With the 15 × 1 reconstruction, only two spheres were visible on the ME 239 keV images and three spheres were visible on the HE 239 keV images. Four and five spheres were visualised on the ME and HE 79 keV images, respectively, again indicating that the images converged with fewer reconstruction updates for the 79 keV window. The equivalent images without a filter can be found in Additional file 1.

**Fig. 9:**
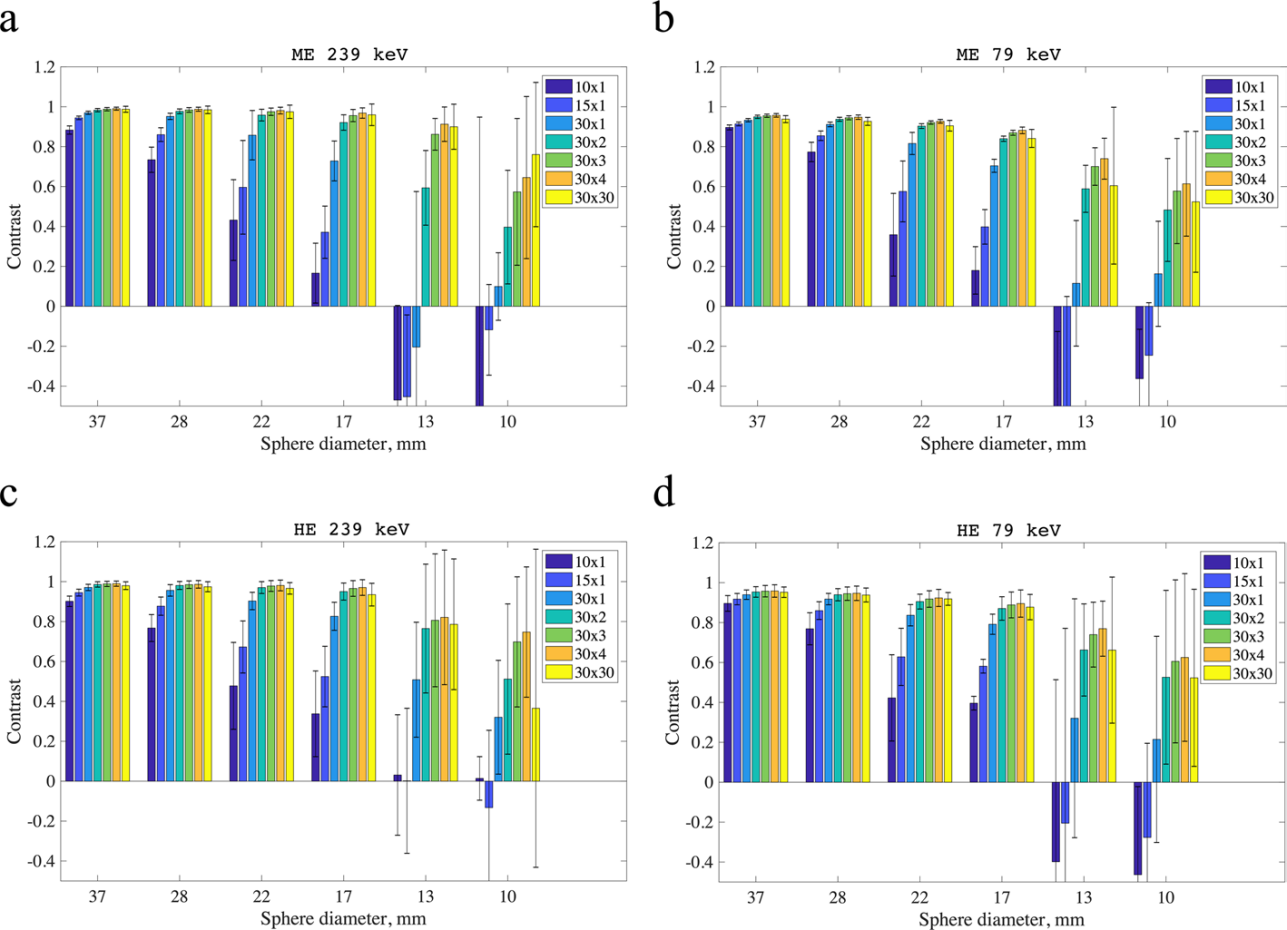
Contrast plotted against sphere diameter for all the image protocols for the different reconstructions with a 12-mm Gaussian filter. Contrast was calculated with Eq. 4, except for when the source VOI included no counts. In cases with no counts in the source VOI, contrast was set to 0 to find averages and SDs. The equivalent plots based on unfiltered images can be found in the supplementary material. The ME 239 keV results are shown in panel **a**, the ME 79 keV results are shown in panel **b**, the HE 239 keV results are shown in panel **c**, and the HE 79 keV results are shown in panel **d**

In Fig. 9, contrast is shown for the four imaging protocols for all filtered reconstructions. The error bars show SDs of measurements repeated at different activity concentrations. As sphere volumes decreased, reconstructions with few reconstruction updates gave poorer contrasts. For all four protocols, the contrast was close to one for the four largest spheres. For the two smallest spheres, the contrast became more variable and error bars became notably larger.

## Discussion

The CFs for ^212^Pb were stable when the total imaged activity exceeded 1 MBq (0.16 kBq/ml) for all four imaging protocols studied with an acquisition time of 30 min. Additionally, it was found that the activity in volumes of 2.6 ml could be quantified with a fractional uncertainty around 20% with ^212^Pb with all protocols except HE 239 keV. This uncertainty includes contributions from the calibration factors and from small volume effects, but other sources of uncertainty, such as from a dose calibrator, were not included. All the imaging protocols had a large increase in fractional uncertainty for the two smallest volumes, and volumes of 1.15 ml were considered too small for reliable activity quantitation with ^212^Pb. When evaluating the visual quality of the images, the 79 keV window gave images which converged with fewer reconstruction updates, but the images were noisier than the 239 keV window images. The HE 79 keV stood out as the visually best protocol due to more visible spheres in the images with a lower activity concentration. There were only small differences in the quantitative uncertainties and visual quality of the protocols, which all showed promise for clinical imaging.

The limit of quantitative ability was determined to be 1 MBq (or 0.16 kBq/ml), based on the CFs no longer being stable. This limit might be lower if the acquisition time is increased. While the limit is based on the total activity in the field of view, it is not a hard limit and smaller total activities with much higher activity concentrations can still be quantified. This can be seen from Fig. 4, where the lowest activity concentration is 17.2 kBq/ml, corresponding to a total activity of 0.8 MBq, but the data points still lie close to the mean. Depending on the treatment (amount of activity administered, biodistribution, biological half-life, etc.), this lower limit can be used to estimate the latest feasible imaging time points in upcoming trial protocols. For example, for the PSMA-targeting ^212^Pb-NG001, if it has a similar biological half-life as PSMA-617, the effective half-life of ^212^Pb-NG001 should be around 8.7 h. The effective half-life with [lutetium-177 (^177^Lu)]Lu-PSMA-617 was calculated to be between 35 and 40 h for most patients [30], giving a biological half-life of around 49 h. As clinically relevant administered activity is likely to be 60–80 MBq [25], there will probably be around 1 MBq ^212^Pb-NG001 left in the patient after 48 h. However, since close to 1 MBq is probably needed in the camera field of view to obtain reliable quantitative results and the activity will be distributed throughout the body, the final imaging time point should be less than 48 h post-injection.

As stable quantitative results can be obtained with around 1 MBq in the field of view, spatial resolution is likely a larger issue than sensitivity, as the primary target for most ^212^Pb-based therapies will be micrometastases. If the tumours are smaller than 2.6 ml, one cannot expect quantitative tumour dosimetry based on these results. However, as long as the volume is larger than 2.6 ml, it should be possible to calculate the activity in organs at risk and larger tumours with a reasonable uncertainty of around 20% from the CFs and small volume effects. The uncertainty did not decrease with increasing volume, and an uncertainty around 20% should be expected for all volumes larger than 2.6 ml. From Fig. 4, it could, however, be seen that the variation in measured to known activity in the spheres increased at lower activity concentrations. Likely, greater accuracy than 20% can be achieved for VOIs with high activity. Performing dosimetry on these volumes is therefore feasible, but factors such as daughter nuclides released from the carrier molecule, the complex dose dependence of relative biological effectiveness, and non-uniform radiation at the micro- and multicellular levels will also add uncertainty to the dosimetry calculations [31–34]. The fractional uncertainties on activity quantitation in spheres were very similar between using mean counts (on the left of Fig. 6) and maximum counts (on the right of Fig. 6), but these results were obtained with the same activity concentration in the entire spherical volume. This is rarely the case in patient tumours and the uncertainties associated with a maximum rather than a mean value are expected to increase.

In Fig. 8, 17–19 kBq/ml is referred to as a low activity concentration. In terms of quantitative imaging, this is a low activity concentration, as it corresponds to a total activity of 0.8 MBq in the phantom, which is just below the limit for stable CFs. 17–19 kBq/ml is likely also a low activity concentration in terms of tumour concentration. For example, for [^177^Lu]Lu-PSMA-617 it has been shown that a typical tumour reaches its maximum activity concentration around 10 h post-injection [35]. Correcting for physical half-lives and assuming similar pharmacokinetic properties of the radiotherapeutics when replacing ^177^Lu with ^212^Pb, this would give an activity concentration around 32 kBq/ml with ^212^Pb after a 100 MBq injection. While there are possibly large variations, low activity images in Fig. 8 might still give an indication of the visibility of a typical tumour at the later imaging time points.

Whether monitoring dose to organs at risk is more important than tumour dosimetry is debatable. Quantitation of tumour absorbed doses are very useful for dose–effect estimates and to avoid undertreating, but doses to organs at risk are important to ensure the safety of the radiopharmaceutical. Kidneys are considered one of the main organs at risk for many radionuclide therapies including ^212^Pb-NG001 [25] and clinical studies with other PSMA-targeting radiopharmaceuticals have also shown salivary glands, liver, and bone marrow as normal tissues at risk [35–37]. Kidney dosimetry should be achievable with ^212^Pb, but potential non-uniform uptake in the kidneys will be difficult to visualise and quantify. Localisation of the radiopharmaceutical in radiosensitive subregions of the kidneys might give whole organ toxicity that is poorly related to mean absorbed kidney dose [38]. This will be difficult to predict solely on the ^212^Pb images, and therefore preclinical data or imaging of surrogate diagnostic compounds in combination with pharmacokinetic models should be used for dosimetry calculations on that level [34, 39].

A minimised uncertainty on the activity quantitation was the primary goal when comparing imaging protocols. For instance, the ME 239 keV protocol had the highest activity recovery and was hence the most resilient against intensity spread, but since this can be accounted for with RCs this characteristic was deemed less substantial. The uncertainties of the curve fits are hence a more relevant property for comparison. Importantly, the uncertainties given in Table 1 reflect the appropriateness of the fitted function, not the quantitative ability of the protocol. The fractional errors on the fitting parameters were smaller for the 239 keV window, but the fractional uncertainties on activity quantitation in spheres were smaller for the 79 keV window. After inspecting the RC curves, it is not surprising that the fits for the 79 keV window are poorer, as the fitted curve seems to reach towards 1 while the data points to a larger extent flatten out. Attenuation plays a larger role for 79 keV photons than for 239 keV photons and is likely what causes the lower activity recovery seen for 79 keV. The photons lost to attenuation in the NEMA phantom have not been accounted for with the CFs, since they were calculated from homogeneously distributed ^212^Pb. When adding *b*_*3*_ as a third fitting parameter to the RC function,

5 $$RC_{{{\text{fit}}}} = b_{3} - \frac{1}{{1 + \frac{v}{{b_{1} }}^{{b_{2} }} }},$$

*b*_*3*_ was less than 1 for all the filtered reconstructions, expect 10 × 1, for all four imaging protocols. For the reconstructions 30 × 2, 30 × 3, and 30 × 4, 1 was more than two SE from *b*_*3*_. Hence, it might be inappropriate to force the RC function to 1. An additional challenge posed by increased attenuation of the lower energy photons is that although the NEMA phantom to some degree simulates a body, it would correspond to a small patient. Thus, it is possible, with the small differences observed, that the 239 keV window would give better quantitative results if the circumference of the NEMA phantom was larger.

Since the quantitative ability was similar between the protocols, visual quality more strongly impacts the choice for clinical imaging. No activity was added to the background when imaging the NEMA phantom, based on the assumption that a high tumour to background contrast will be seen in patients treated with ^212^Pb-NG001, as has been observed in imaging studies with gallium-68-labelled PSMA [40]. For both energy windows, false positives appeared with increased numbers of updates, which can be regarded more concerning than the noise seen in the images. This may suggest making reconstructions tailored to the patient-specific activity concentration and distribution. A large amount of statistical noise was expected in ^212^Pb images due to the limited number of photons and the large amount of scatter [41] from the ^208^Tl emission of 2.6 MeV. Filters compensate for the noise from scatter and low count statistics; the filtered ^212^Pb images being of higher visual quality were therefore expected. Since the quantitative uncertainties were similar in the filtered and unfiltered images, filtering is recommended. Also, since no large quantitative differences were found, only a 12-mm filter was investigated in this study and the filter strength was not optimised. Likely different reconstructions benefit from different filters. In general, the uncertainties found decreased with increasing number of updates. This, in addition to recovery curves not converging with few iterative updates encourages a higher number of reconstruction updates than what is typical in the clinic. If the same images are used for quantitation and visual interpretation, this might also motivate stronger filters.

The question of whether ^212^Pb can be quantitatively imaged was investigated in this work. As far as we know, no previously published article has undertaken this problem, but an abstract was published in 2019 which compared imaging of one sphere of ^212^Pb with an ME collimator to simulations [42]. The authors concluded that imaging of ^212^Pb could be feasible, but features such as intensity spread in small volumes were not discussed. However, quantitative imaging of α-particle emitter ^223^Ra has been extensively studied. With planar images a 200 ml volume was quantified with 10% uncertainty and a 0.5 ml volume with 40% uncertainty [4]. A SPECT study with ^223^Ra used an ME collimator, a 5/8″ crystal, 2 iterations and 10 subsets with a Butterworth filter and added three energy windows (40% centred on 85 keV, 20% on 154 keV, and 20% on 270 keV). Similarly to our findings with ^212^Pb, they achieved quantitation of 5.6 ml volumes with an error smaller than 19% [43]. The count rate achieved with ^212^Pb with the 79 keV energy window was much higher than when imaging ^223^Ra [4, 6, 13, 43]. However, a lot of photons contributing to the 79 keV energy peak are characteristic X-rays from the lead collimator and scattered photons from the 2.6 MeV emission of ^208^Tl, and hence it would be more relevant to report the number of primary photons detected to compare sensitivity. Still, almost 25 times more activity is expected to be administered with ^212^Pb compared to ^223^Ra therapy, and it is therefore expected that images of reasonable quality can be obtained. Count rates with ^227^Th and ^225^Ac were lower than with ^223^Ra and hence also lower than with ^212^Pb [13]. Other studies have attempted to quantify ^227^Th and ^223^Ra in combination with planar imaging and found that ^227^Th can be separated from ^223^Ra in the images. Spatial filtering improved the images visually, similarly to ^212^Pb [5]. Using a spectral analysis technique modelling energy spectra and scattered photons, Murray et al. calculated that the average difference between known and estimated activity was 5.1% for ^227^Th and 3.4% for ^223^Ra, but differences of 50% were observed [6]. However, even with measurements differing from the known activity with 50%, they obtained differences smaller than 10% when calculating the time integrated activity. Hence, they found that for dosimetry, “uncertainty in individual data measurements may be mitigated by carrying out multiple measurements over several time points” [6]. This is likely also applicable to ^212^Pb, but will require more imaging time points than usually acquired for clinical dosimetry.

## Conclusion

In conclusion, calibration factors for quantitative imaging of ^212^Pb were stable when the total imaged activity exceeded 1 MBq. All imaging protocols studied showed promise for quantitative imaging. For example, for three of the four protocols the fractional uncertainties for the activity quantitation in the 2.6-ml sphere ranged from 16 to 21% for the 30 × 4 filtered reconstruction, accounting for uncertainties introduced by the calibration factors and intensity spread in small volumes. Regardless of the protocol used, the lack of convergence of recovery curves for few reconstruction iterations and decreasing CVs with increasing reconstruction updates encourage reconstructions with a high number of updates. Visual quality at low activity concentrations was better with the 79 keV peak and HE collimator for the geometry investigated, a phantom with spheres. Overall, the results indicate that it could be possible to perform patient-specific dosimetry with SPECT/CT imaging.

## Supplementary Information

**Additional file 1:** All supplementary materials with results from reconstructions not shown in the main paper are found in Additional file 1. Pages 1–12 include all materials from the reconstructions with a 12-mm Gaussian filter applied which were not included in the paper. Pages 13–34 include all materials from the reconstructions without filters applied. Calibration factors for all reconstructions are given in the tables on pages 1 (filtered) and 13 (unfiltered). The equivalent plots as that shown in Fig. 2 **c** are shown for the other reconstructions on page 2 (filtered) and pages 14–15 (unfiltered). The unfiltered versions of Fig. 3 **a** and **b** are found on page 13. Versions of Fig. 4 for other reconstructions are shown on pages 3–5 (filtered) and 15–18 (unfiltered). Recovery plots as shown in Fig. 5 and tables equivalent to Table 1 for other reconstructions are given on pages 6–11 (filtered) and 19–29 (unfiltered). The equivalent plots as those given in Fig. 6 without filters are given on pages 30–31 and the filtered version for the smallest sphere is given on page 12. The unfiltered versions of the images in Fig. 7 and Fig. 8 are shown on page 32 and 33, respectively. The version of Fig. 9 without a filter is given on page 34.

## Data Availability

Freely available upon request.

## Abbreviations

α: Alpha
β: Beta
^212^Pb: Lead-212
^223^Ra: Radium-223
^212^Bi: Bismuth-212
^208^Tl: Thallium-208
^227^Th: Thorium-227
^213^Bi: Bismuth-213
^225^Ac: Actinium-225
^149^Tb: Terbium-149
^203^Pb: Lead-203
^177^Lu: Lutetium-177
SPECT: Single photon-emission computed tomography
CT: Computed tomography
PET: Positron emission tomography
CF: Calibration factor
RC: Recovery coefficient
CV: Coefficient of variation
PSMA: Prostate-specific membrane antigen
TAT: Targeted alpha therapy
HE: High energy
ME: Medium energy
NEMA: National Electrical Manufacturers Association
IEC: International Electrotechnical Commision
VOI: Volume of interest
SD: Standard deviation
SE: Standard error
