# Correction to: Quantitative SPECT/CT imaging of lead-212: a phantom study

**DOI:** 10.1186/s40658-022-00499-3

**Published:** 2022-10-10

**Authors:** Monika Kvassheim, Mona-Elisabeth R. Revheim, Caroline Stokke

**Affiliations:** 1 Department of Physics and Computational Radiology, Division of Radiology and Nuclear Medicine, Oslo University Hospital, Oslo, Norway; 2 Faculty of Medicine, University of Oslo, Oslo, Norway; 3 Department of Nuclear Medicine, Division of Radiology and Nuclear Medicine, Oslo University Hospital, Oslo, Norway; 4 Department of Physics, University of Oslo, Oslo, Norway

## Correction to: EJNMMI Physics 9, 52 (2022) 10.1186/s40658-022-00481-z

Following publication of the original article [1], an error in Fig. 1 was reported by the authors. In the top blue box, the intensity of the 87.1 keV X-ray was quoted as 1.2%, but it should be 2.0%. The updated figure is provided in this correction article.

The authors would also like to specify that a scatter filter of 12 mm was used for all the SPECT images analysed. The ‘no filter’ results refer to images where no post-filter was applied.

The original article [1] has been updated.

**Fig. 1:**
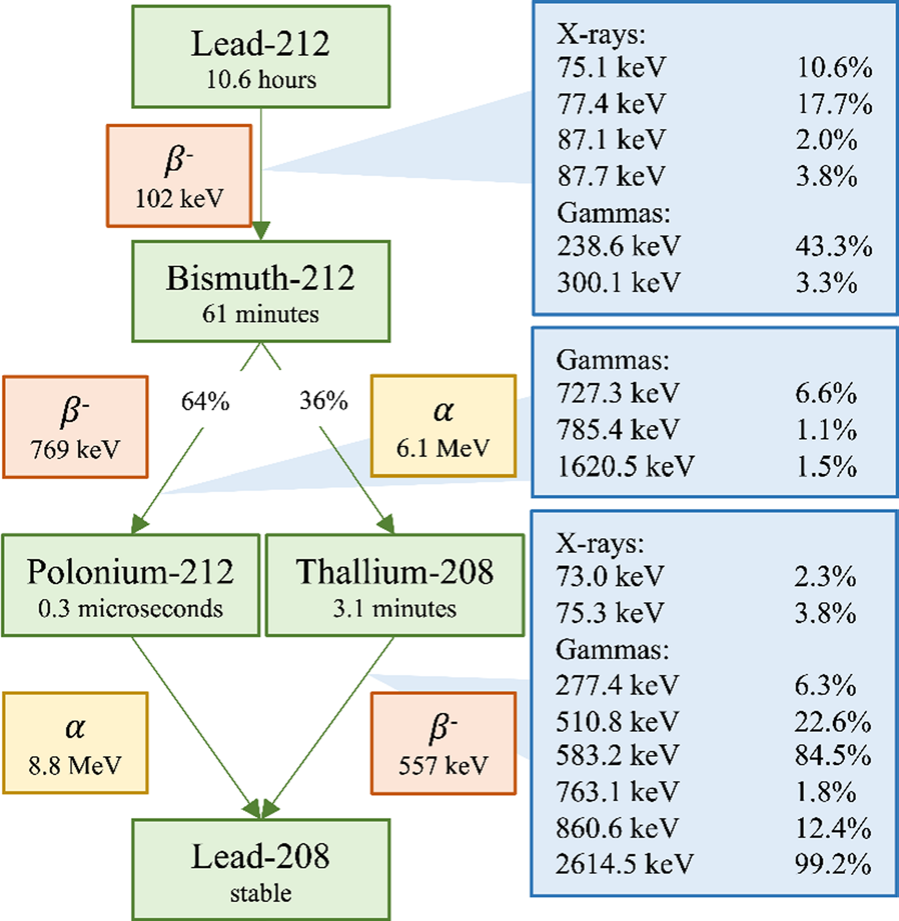
Decay scheme of 212Pb. The mean alpha and beta energies are included. The relevant photons emitted and their energies are added to the transition. Photons with emission probabilities smaller than 1% per 212Pb decay or with energies below 70 keV are not included. The data are taken from ICRP 107 [2]
