# Supplementary material for: Quantitative SPECT/CT imaging of lead-212: a phantom study
Source: EJNMMI Phys. 2022 Aug 4;9:52. doi: 10.1186/s40658-022-00481-z (PMC9352840; doi:10.1186/s40658-022-00481-z)

Supplementary material

Supplementary figures and tables for images filtered with a 12 mm Gaussian filter:

Calibration factors [cps/kBq], 12 mm Gaussian filter:

Calibration factors [cps/kBq] for the four imaging protocols for all the filtered reconstructions with standard deviations.

	**10x1**	**15x1**	**30x1**	**30x2**	**30x3**	**30x4**	**30x30**
**ME 79 keV**	0.38 ±0.05	0.45 ±0.07	0.50 ±0.07	0.51 ±0.06	0.51 ±0.07	0.51 ±0.07	0.50 ±0.05
**HE 79 keV**	0.28 ±0.07	0.31 ±0.06	0.34 ±0.04	0.34 ±0.04	0.34 ±0.04	0.34 ±0.04	0.34 ±0.05
**ME 239 keV**	0.05 ±0.03	0.05 ±0.03	0.06 ±0.02	0.07 ±0.02	0.08 ±0.01	0.08 ±0.01	0.08 ±0.01
**HE 239 keV**	0.07 ±0.03	0.07 ±0.02	0.07 ±0.02	0.08 ±0.02	0.08 ±0.02	0.08 ±0.02	0.08 ±0.02

Calibration factor plots, 12 mm Gaussian filter:

The datapoints for calibration factors plotted against activity for the four imaging protocols for all the filtered reconstructions.

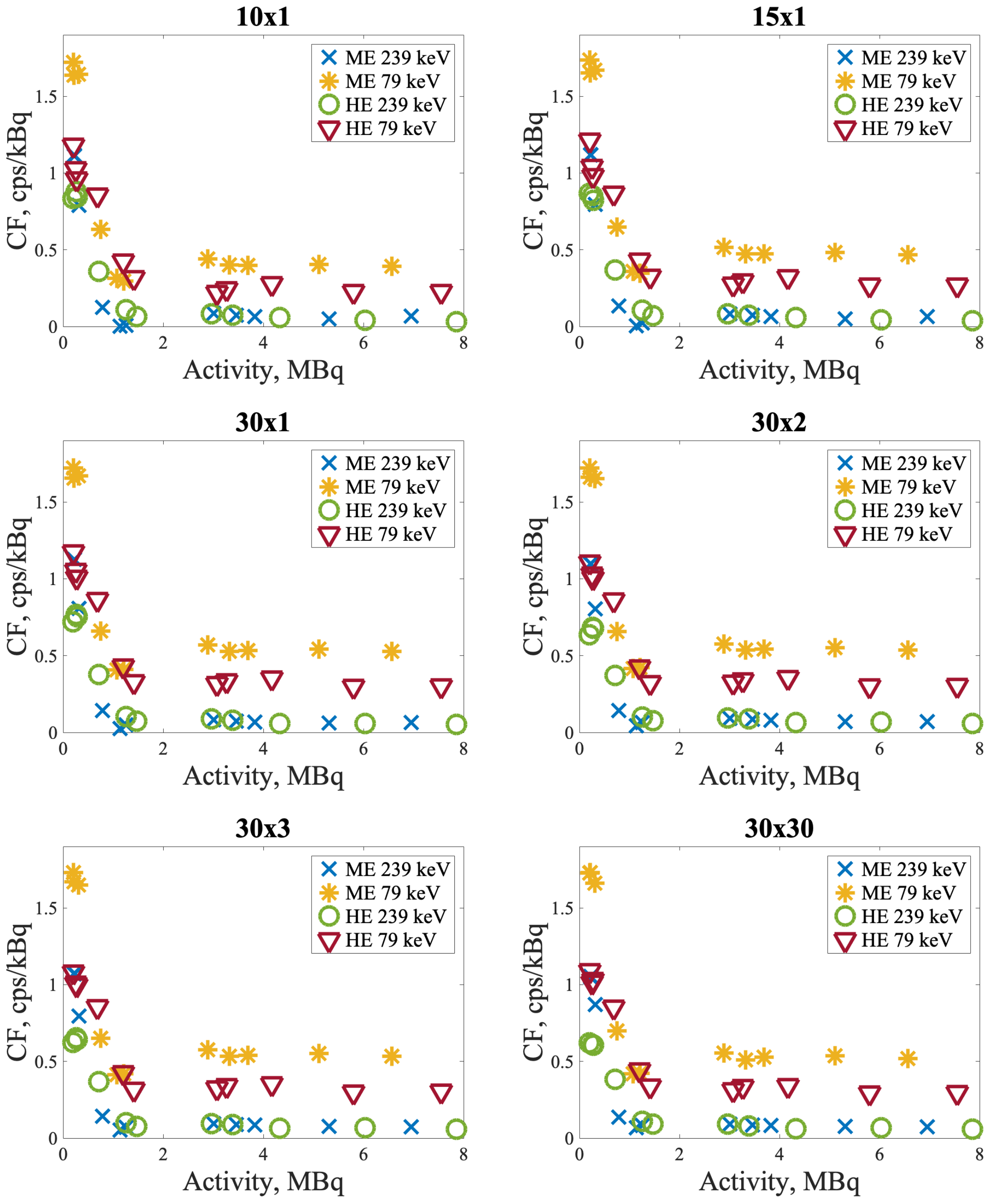

Measured activity in the spheres by physical activity in the spheres with a 12 mm Gaussian filter applied:

The measured activity in each sphere divided by the known, physical activity in each sphere plotted against activity concentration. The figure shows the results found with all filtered reconstructions for the different imaging protocols. The mean value for each sphere volume is shown as a line. Note that the x-axes are the same, but the values on the y-axes differ.

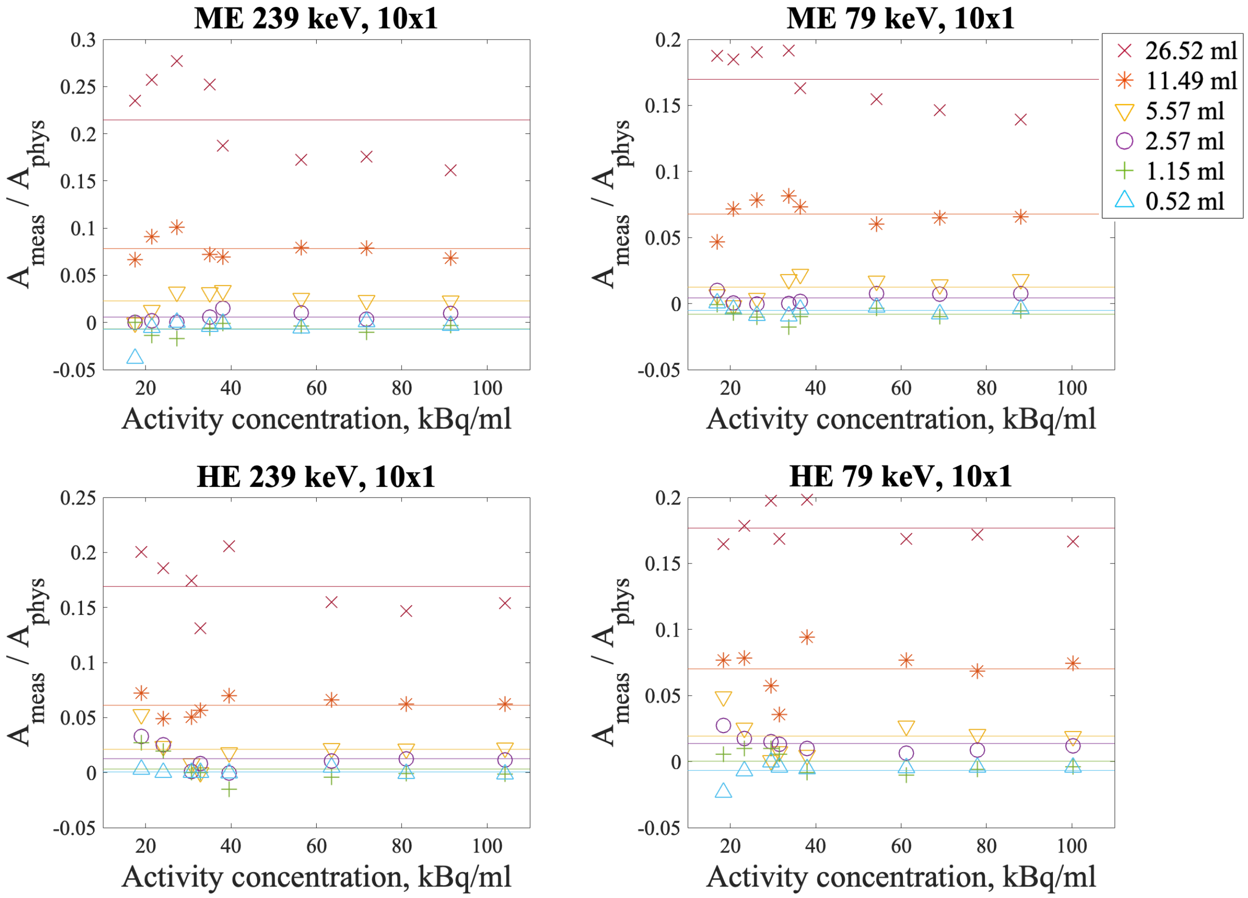
ME 239 keV, 10x1
ME 79 keV, 10x1
HE 239 keV, 10x1
HE 79 keV, 10x1
A_{meas} / A_{phys}
Activity concentration, kBq/ml
26.52 ml
11.49 ml
5.57 ml
2.57 ml
1.15 ml
0.52 ml

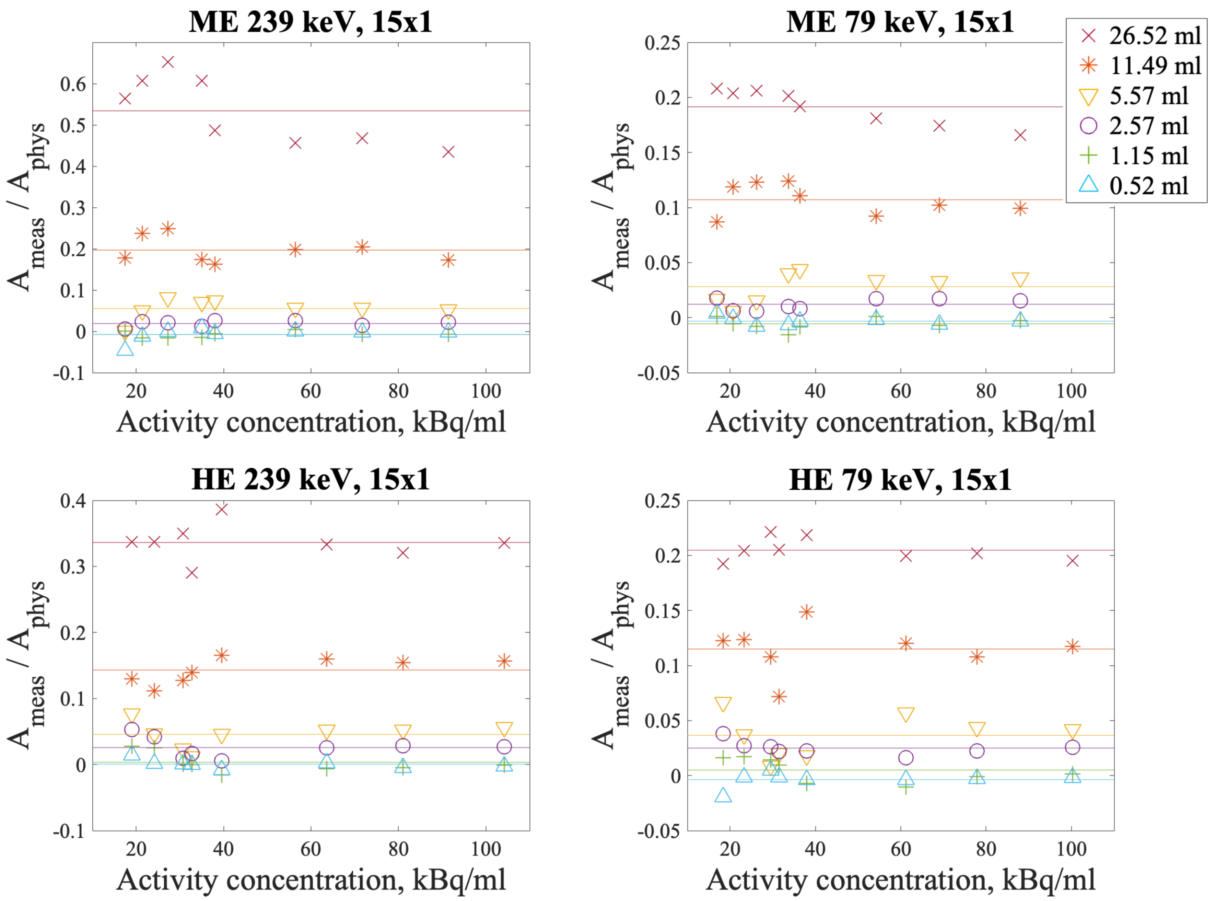
ME 239 keV, 15x1
ME 79 keV, 15x1
HE 239 keV, 15x1
HE 79 keV, 15x1
A_{meas} / A_{phys}
Activity concentration, kBq/ml
26.52 ml
11.49 ml
5.57 ml
2.57 ml
1.15 ml
0.52 ml

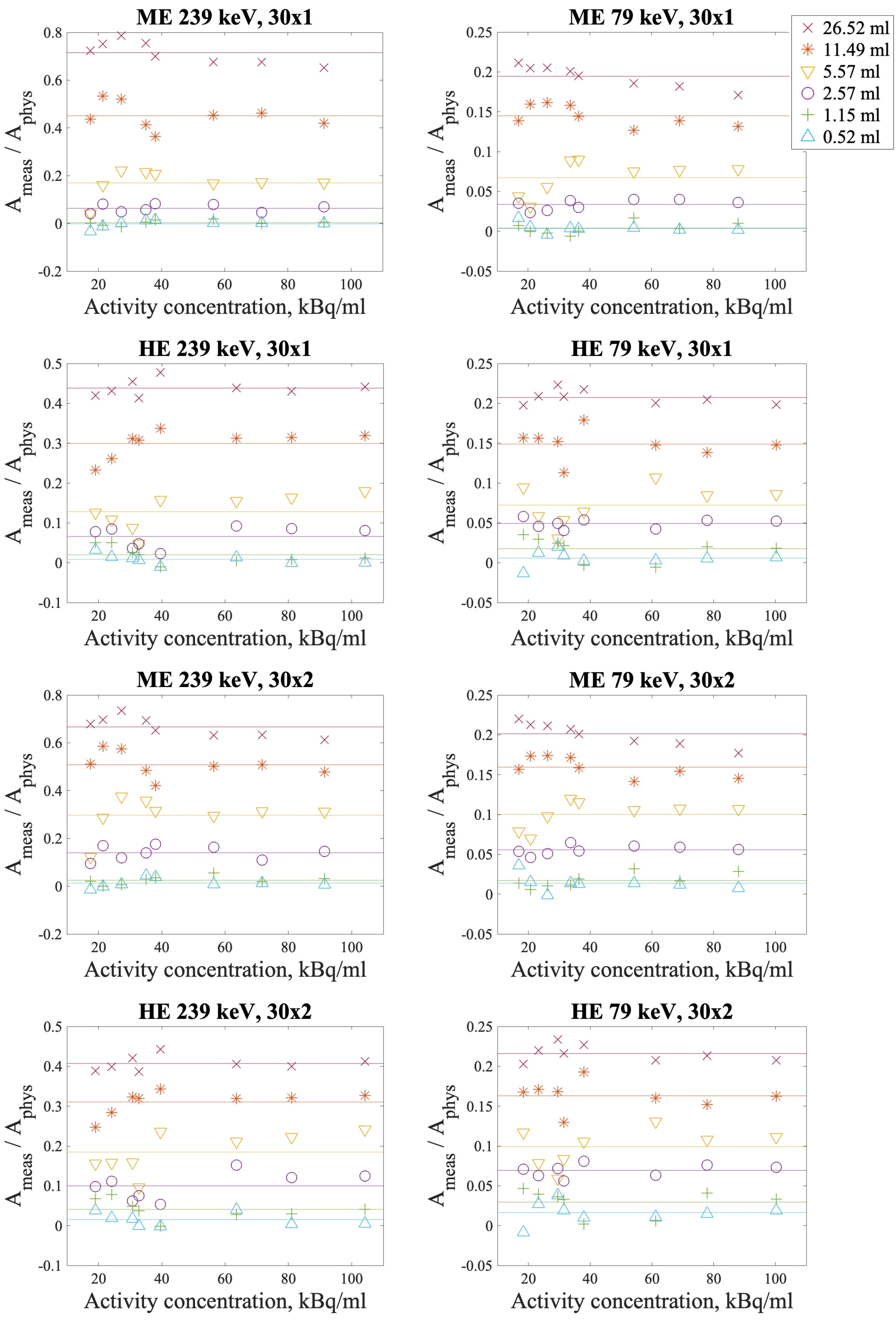
ME 239 keV, 30x1
ME 79 keV, 30x1
HE 239 keV, 30x1
HE 79 keV, 30x1
ME 239 keV, 30x2
ME 79 keV, 30x2
HE 239 keV, 30x2
HE 79 keV, 30x2
A_{meas} / A_{phys}
Activity concentration, kBq/ml
26.52 ml
11.49 ml
5.57 ml
2.57 ml
1.15 ml
0.52 ml

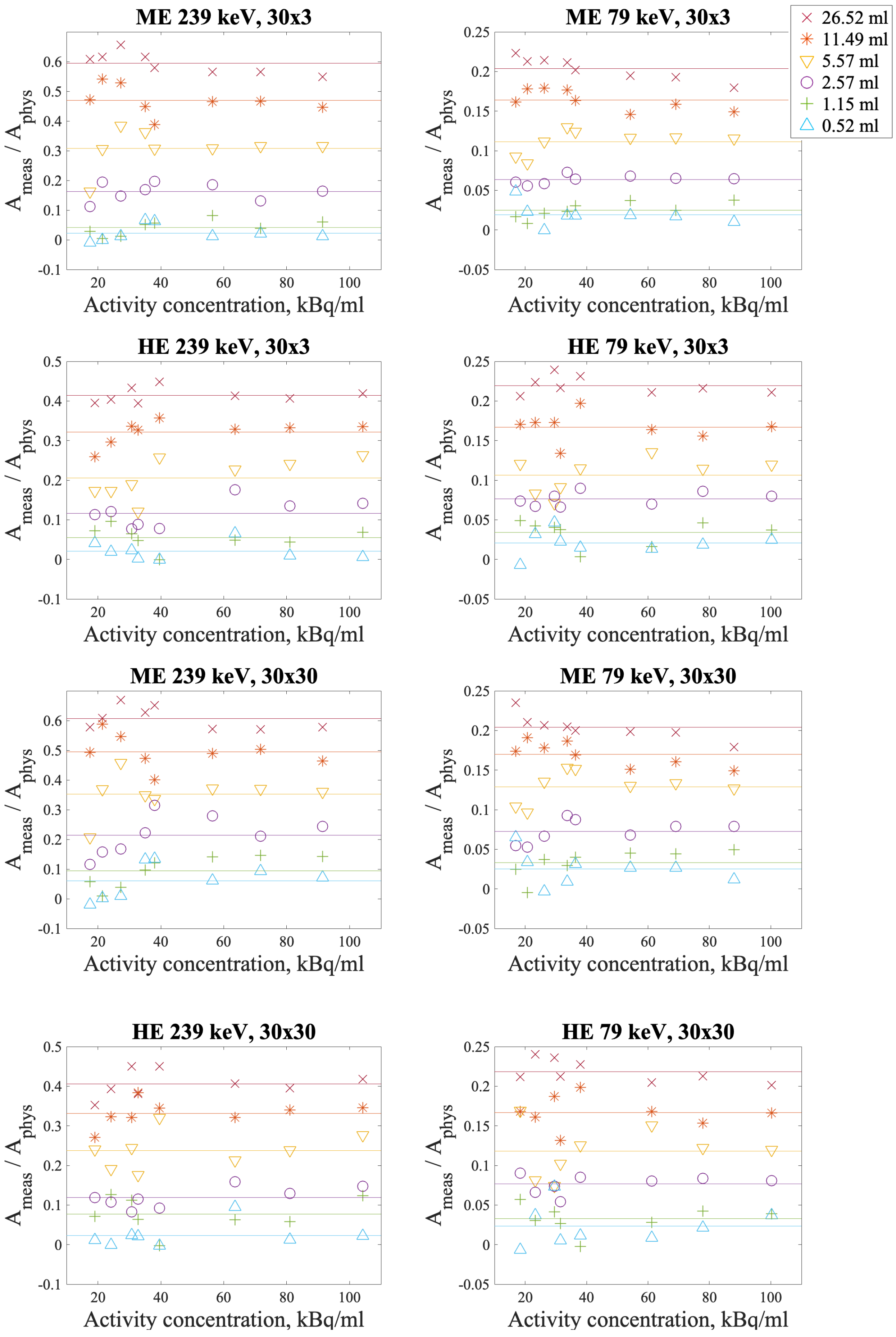
ME 239 keV, 30x3
ME 79 keV, 30x3
HE 239 keV, 30x3
HE 79 keV, 30x3
ME 239 keV, 30x30
ME 79 keV, 30x30
HE 239 keV, 30x30
HE 79 keV, 30x30
A_{meas} / A_{phys}
Activity concentration, kBq/ml
26.52 ml
11.49 ml
5.57 ml
2.57 ml
1.15 ml
0.52 ml

RC fits for all filtered reconstructions:

RC_{mean} and RC_{max} plotted for the four imaging protocols for all the filtered reconstructions. Curve fits applied to RC_{mean} are also plotted. The fitting parameters and their associated uncertainties are presented in tables for each reconstruction. Note that the y-axes differ.

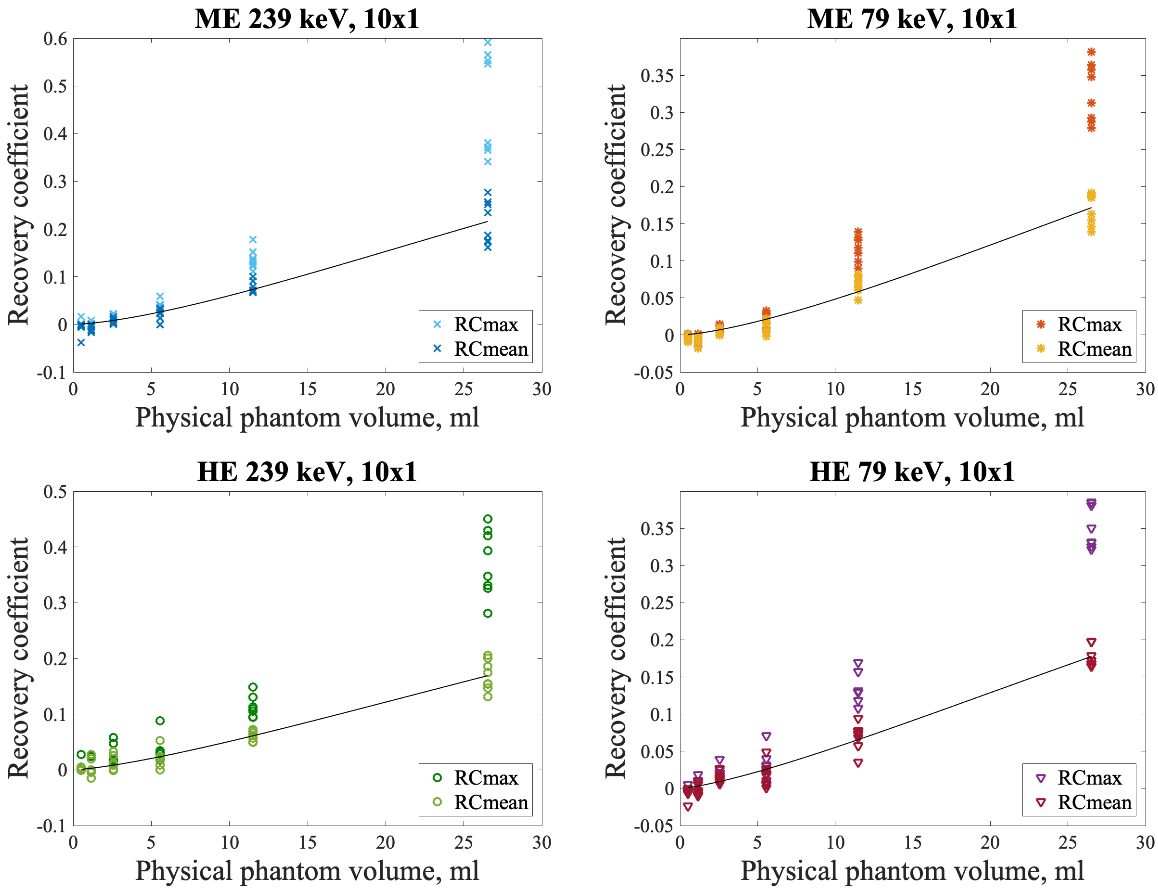

RC_{mean}, 10x1	Parameter	Value	Standard error	Fractional standard error
**ME 239 keV**	b_1	62.99 ml	5.066 ml	8%
	b_2	1.491	0.111	7%
**ME 79 keV**	b_1	79.02 ml	5.903 ml	7%
	b_2	1.441	0.084	6%
**HE 239 keV**	b_1	85.31 ml	7.707 ml	9%
	b_2	1.363	0.089	7%
**HE 79 keV**	b_1	83.12 ml	6.176 ml	7%
	b_2	1.341	0.073	5%

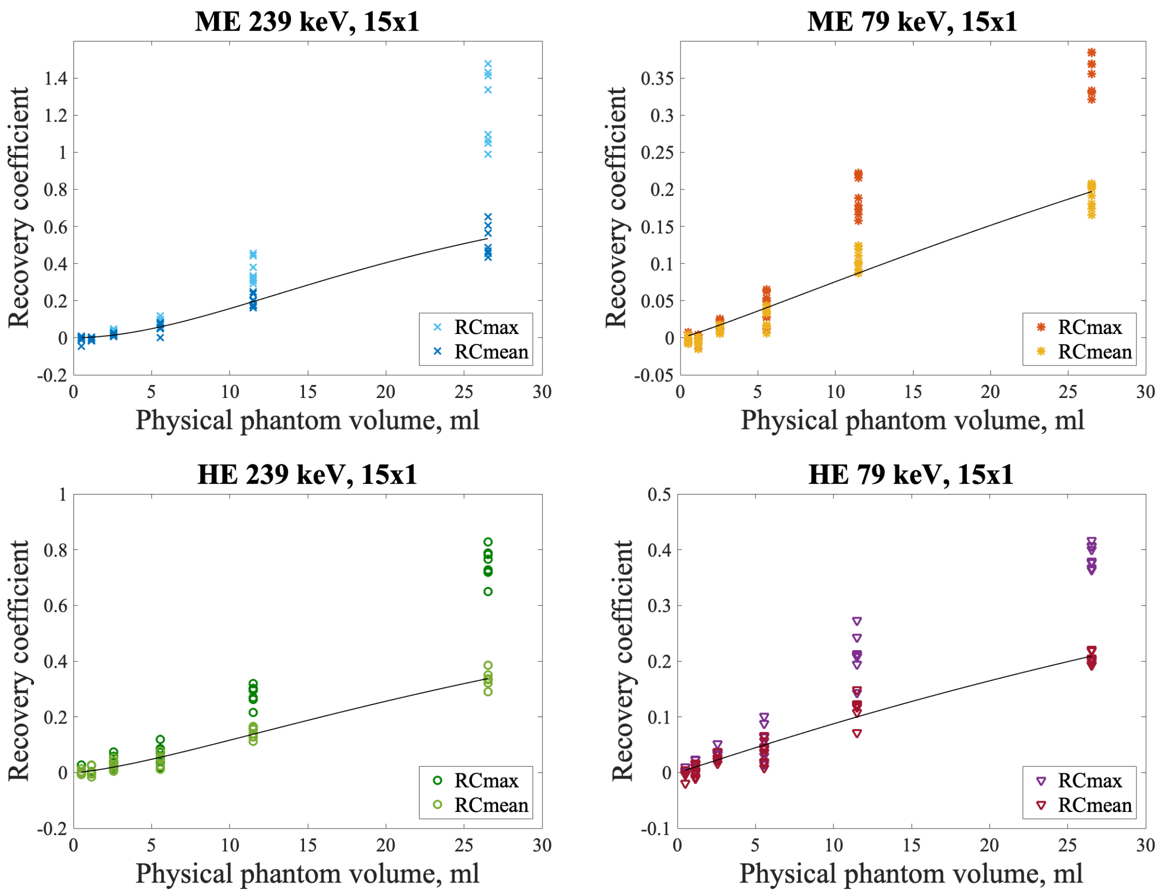

RC_{mean}, 15x1	**Parameter**	**Value**	**Standard error**	**Fractional standard error**
**ME 239 keV**	b_1	24.61 ml	0.658 ml	3%
	b_2	1.865	0.099	5%
**ME 79 keV**	b_1	92.19 ml	8.222 ml	9%
	b_2	1.127	0.064	6%
**HE 239 keV**	b_1	43.11 ml	1.492 ml	3%
	b_2	1.388	0.055	4%
**HE 79 keV**	b_1	95.47 ml	8.582 ml	9%
	b_2	1.039	0.0556	5%

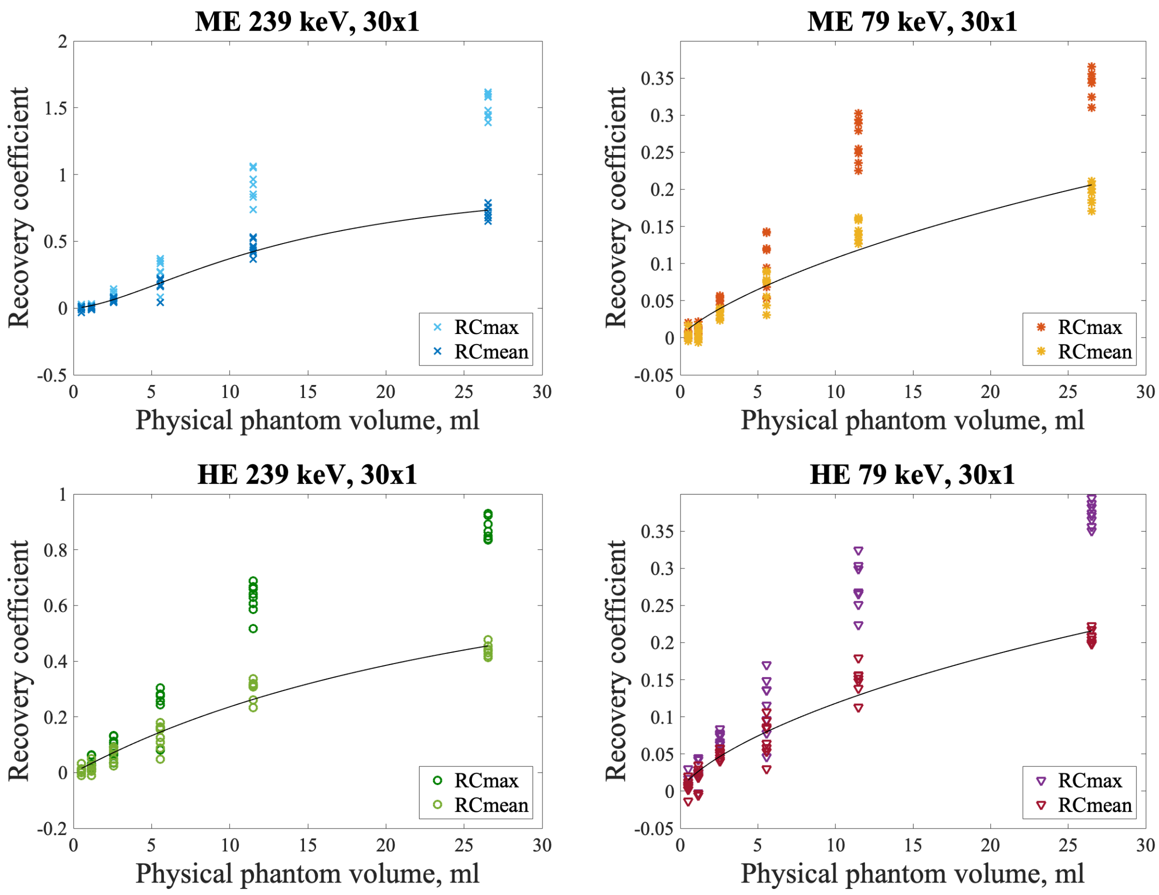

RC_{mean}, 30x1	**Parameter**	**Value**	**Standard error**	**Fractional standard error**
**ME 239 keV**	b_1	14.00 ml	0.383 ml	3%
	b_2	1.583	0.068	4%
**ME 79 keV**	b_1	146.3 ml	19.898 ml	14%
	b_2	0.7895	0.047	6%
**HE 239 keV**	b_1	31.70 ml	1.60 ml	5%
	b_2	1.018	0.0496	5%
**HE 79 keV**	b_1	152.4 ml	19.46 ml	13%
	b_2	0.7381	0.0392	5%

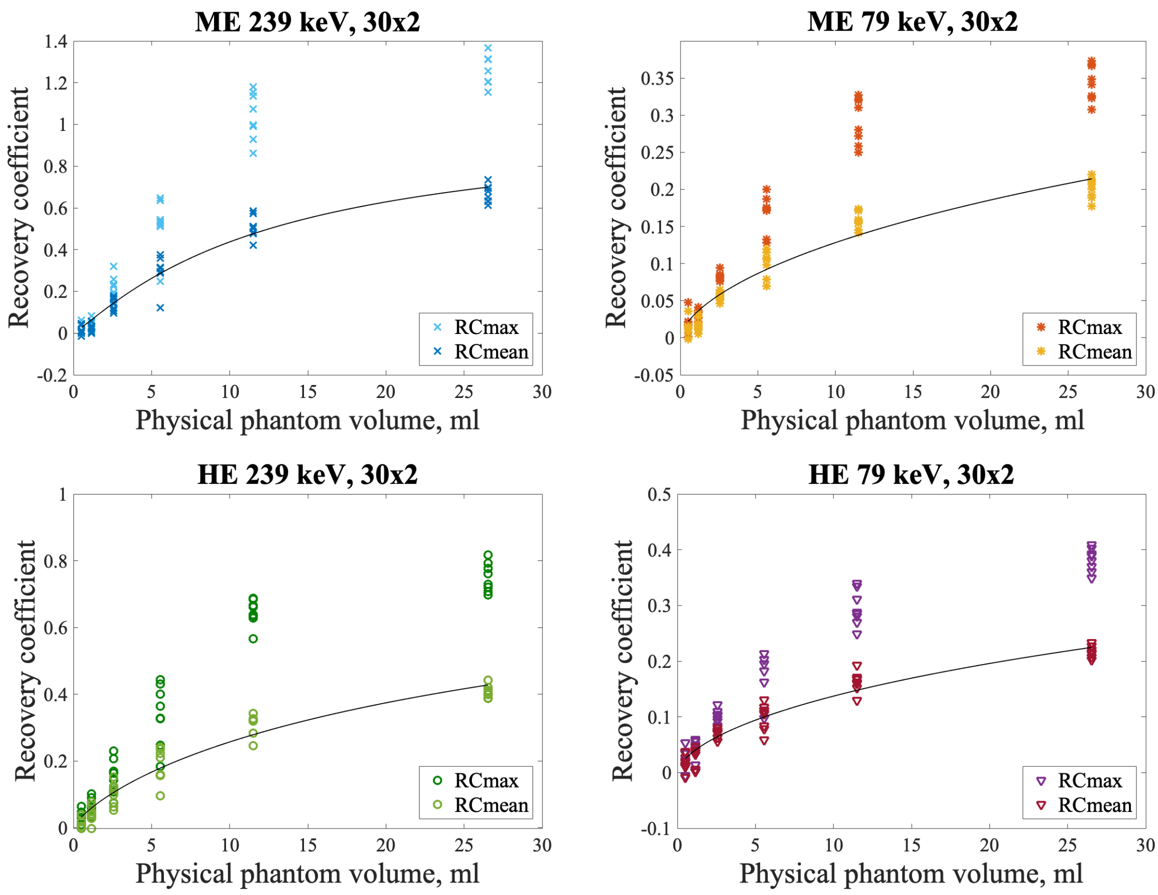

RC$_{mean}$, 30x2	**Parameter**	**Value**	**Standard error**	**Fractional standard error**
**ME 239 keV**	b_1	12.52 ml	0.5179 ml	4%
	b_2	1.125	0.055	5%
**ME 79 keV**	b_1	206.7 ml	30.689 ml	15%
	b_2	0.633	0.033	5%
**HE 239 keV**	b_1	38.34 ml	2.824 ml	7%
	b_2	0.7897	0.0412	5%
**HE 79 keV**	b_1	199.2 ml	27.96 ml	14%
	b_2	0.6145	0.0303	5%

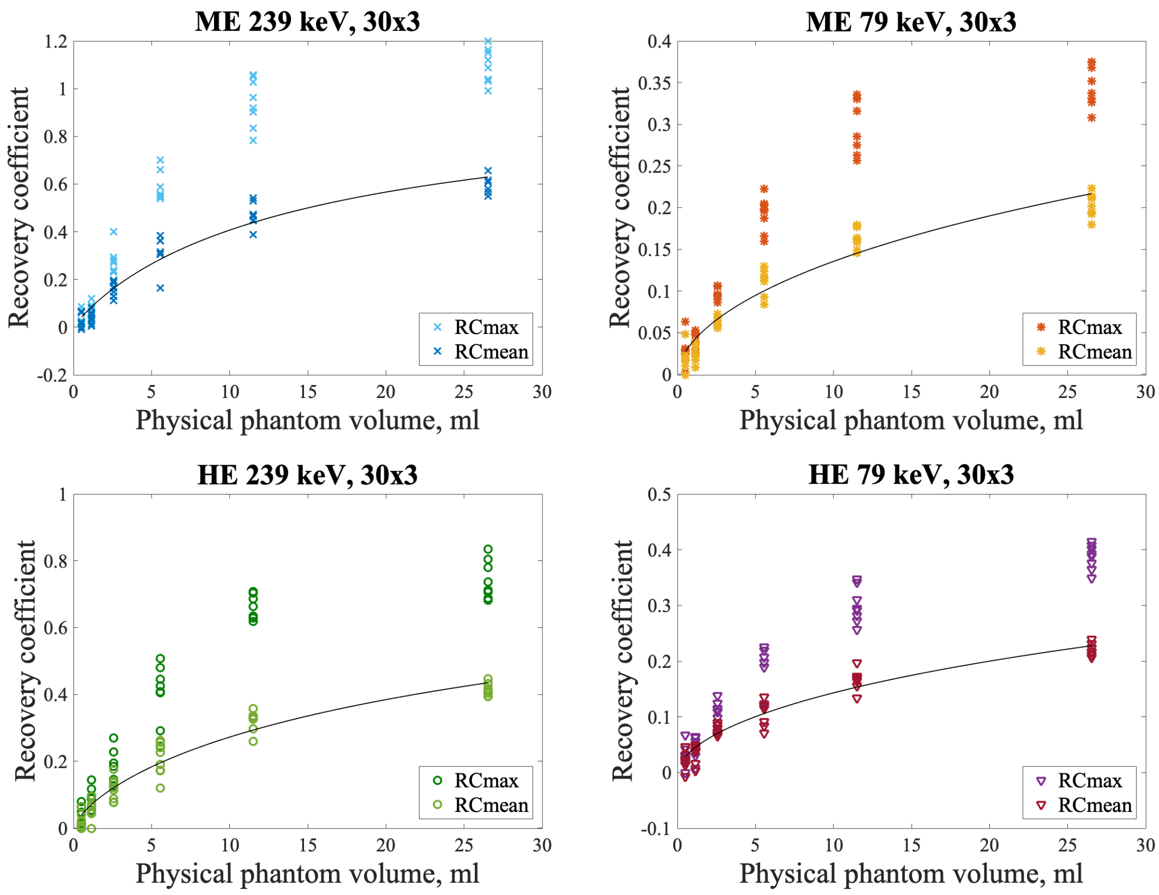

RC_{mean}, 30x3	**Parameter**	**Value**	**Standard error**	**Fractional standard error**
**ME 239 keV**	b_1	15.00 ml	0.7526 ml	5%
	b_2	0.9285	0.0471	5%
**ME 79 keV**	b_1	240.3 ml	37.526 ml	16%
	b_2	0.5828	0.0297	5%
**HE 239 keV**	b_1	37.79 ml	2.893 ml	8%
	b_2	0.7384	0.0380	5%
**HE 79 keV**	b_1	216.0 ml	30.791 ml	14%
	b_2	0.5825	0.0279	5%

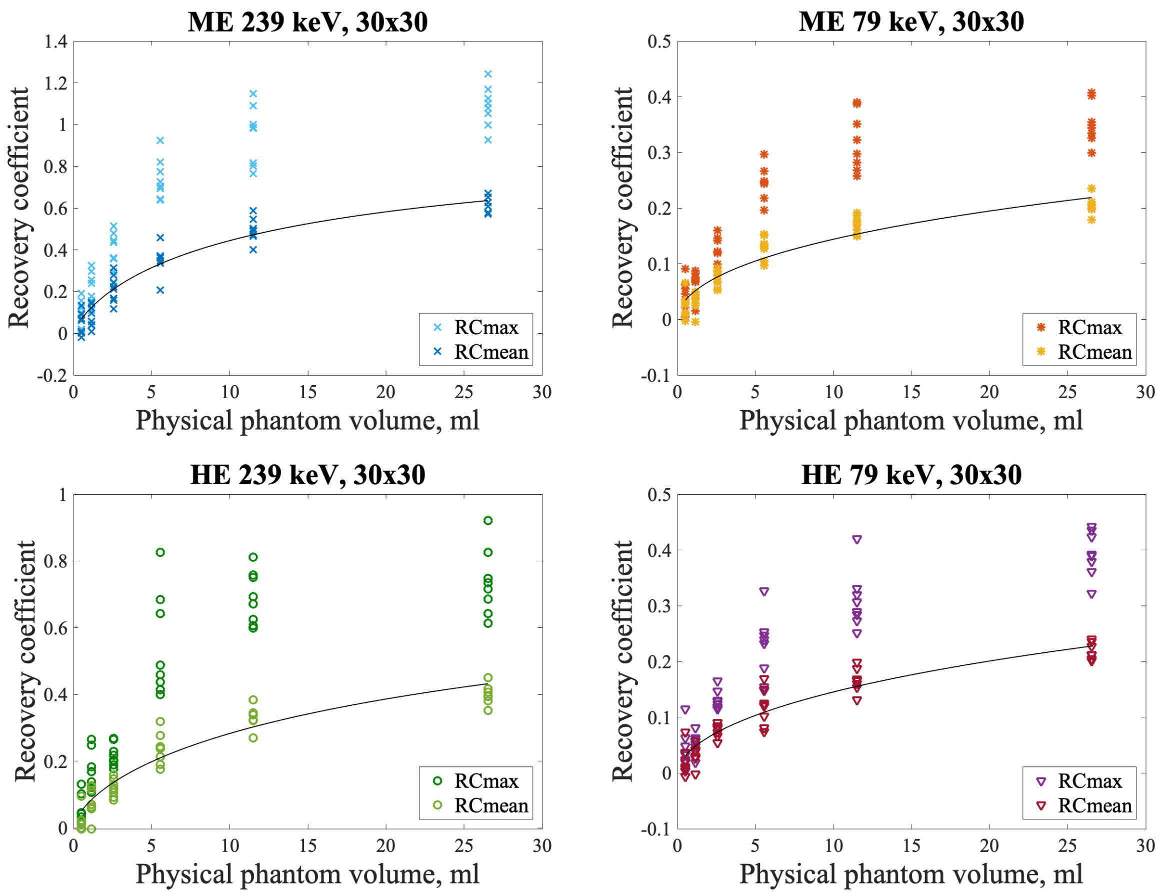

RC_{mean}, 30x30	**Parameter**	**Value**	**Standard error**	**Fractional standard error**
**ME 239 keV**	b_1	13.25 ml	0.8673 ml	7%
	b_2	0.8011	0.0477	6%
**ME 79 keV**	b_1	302.4 ml	65.102 ml	22%
	b_2	0.5229	0.0332	6%
**HE 239 keV**	b_1	39.75 ml	3.913 ml	10%
	b_2	0.673	0.0407	6%
**HE 79 keV**	b_1	232.7 ml	44.362 ml	19%
	b_2	0.5626	0.0348	6%

Fractional uncertainties on RCs for the smallest sphere with a 12 mm Gaussian filter applied:

Fractional uncertainties on activity quantitation in small volumes using mean counts (left) and using maximum counts (right) plotted for the four imaging protocols against reconstruction updates for the smallest spheres on filtered images. The plots for this sphere was not included in the paper as the uncertainties all exceed 60%.

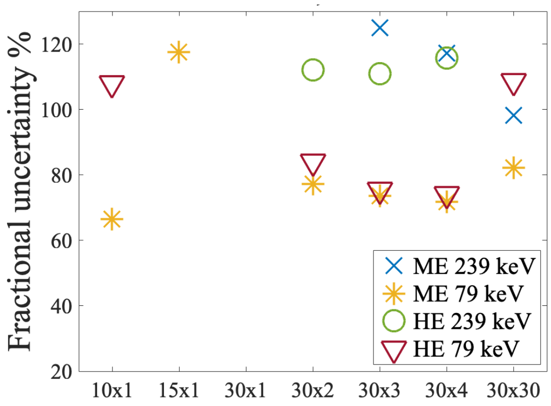

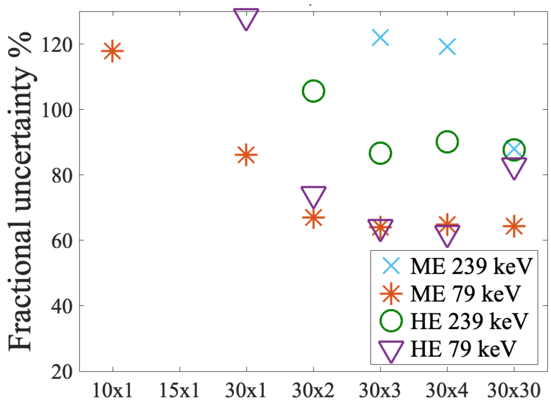

Supplementary figures and tables for images without a filter applied:

Calibration factors [cps/kBq], no filter:

Calibration factors [cps/kBq] for the four imaging protocols for all the unfiltered reconstructions with standard deviations.

	10x1	15x1	30x1	30x2	30x3	30x4	30x30
**ME 79 keV**	0.38 ±0.06	0.45 ±0.07	0.52 ±0.06	0.53 ±0.06	0.53 ±0.06	0.52 ±0.06	0.51 ±0.05
**HE 79 keV**	0.28 ±0.03	0.32 ±0.03	0.35 ±0.03	0.35 ±0.03	0.34 ±0.03	0.34 ±0.03	0.34 ±0.03
**ME 239 keV**	0.05 ±0.03	0.05 ±0.02	0.06 ±0.01	0.07 ±0.01	0.08 ±0.01	0.08 ±0.01	0.08 ±0.01
**HE 239 keV**	0.06 ±0.02	0.07 ±0.02	0.07 ±0.01	0.08 ±0.01	0.08 ±0.01	0.08 ±0.01	0.08 ±0.02

Calibration factors plotted against reconstructions and the associated coefficient of variation for unfiltered images:

To the left, the CFs for unfiltered are plotted for the different reconstructions for the four imaging protocols. The error bars show the standard deviations. To the right the coefficients of variation are plotted for the four imaging protocols for all the reconstructions.

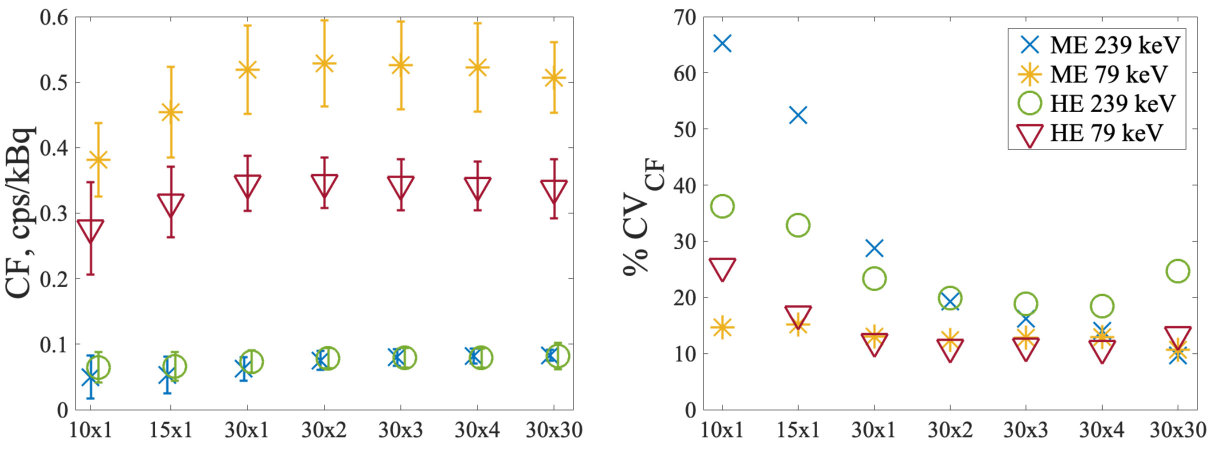

Calibration factor plots, no filter:

The datapoints for calibration factors plotted against activity for the four imaging protocols for all the unfiltered reconstructions.

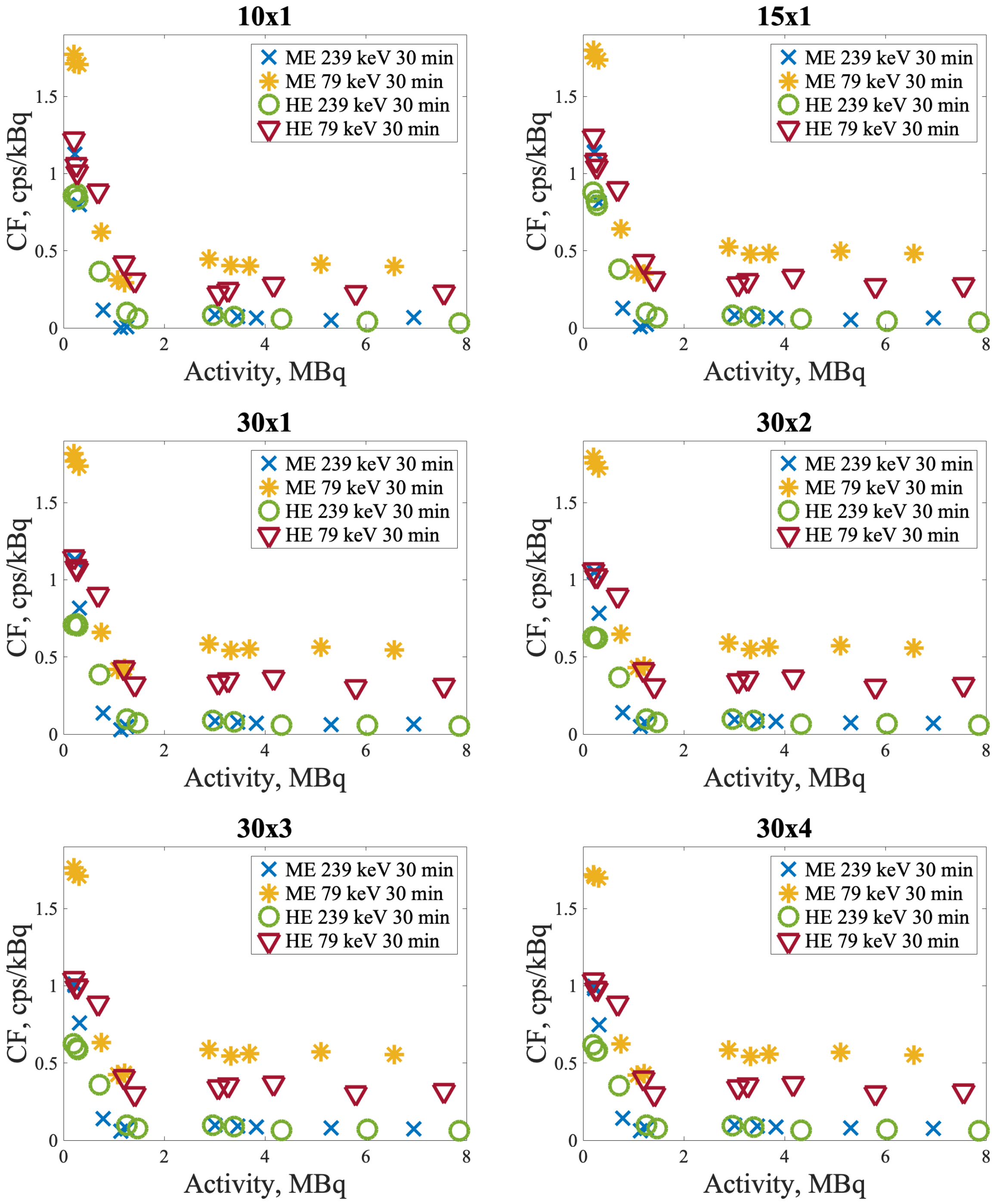

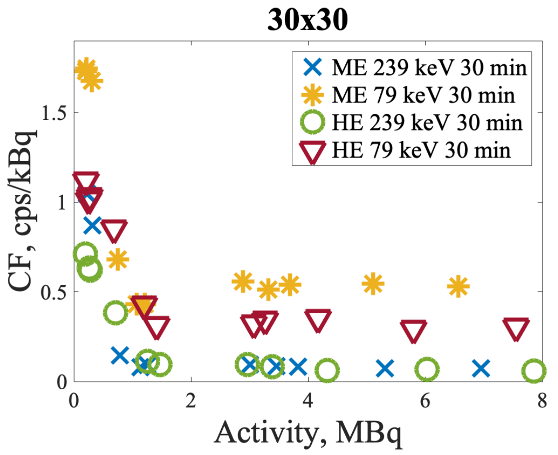

Measured activity in the spheres by physical activity in the spheres for unfiltered images: The measured activity in each sphere divided by the known, physical activity in each sphere plotted against activity concentration. The figure shows the results found with all unfiltered reconstructions for the different imaging protocols. The mean value for each sphere volume is shown as a line. Note that the x-axes are the same, but the values on the y-axes differ.

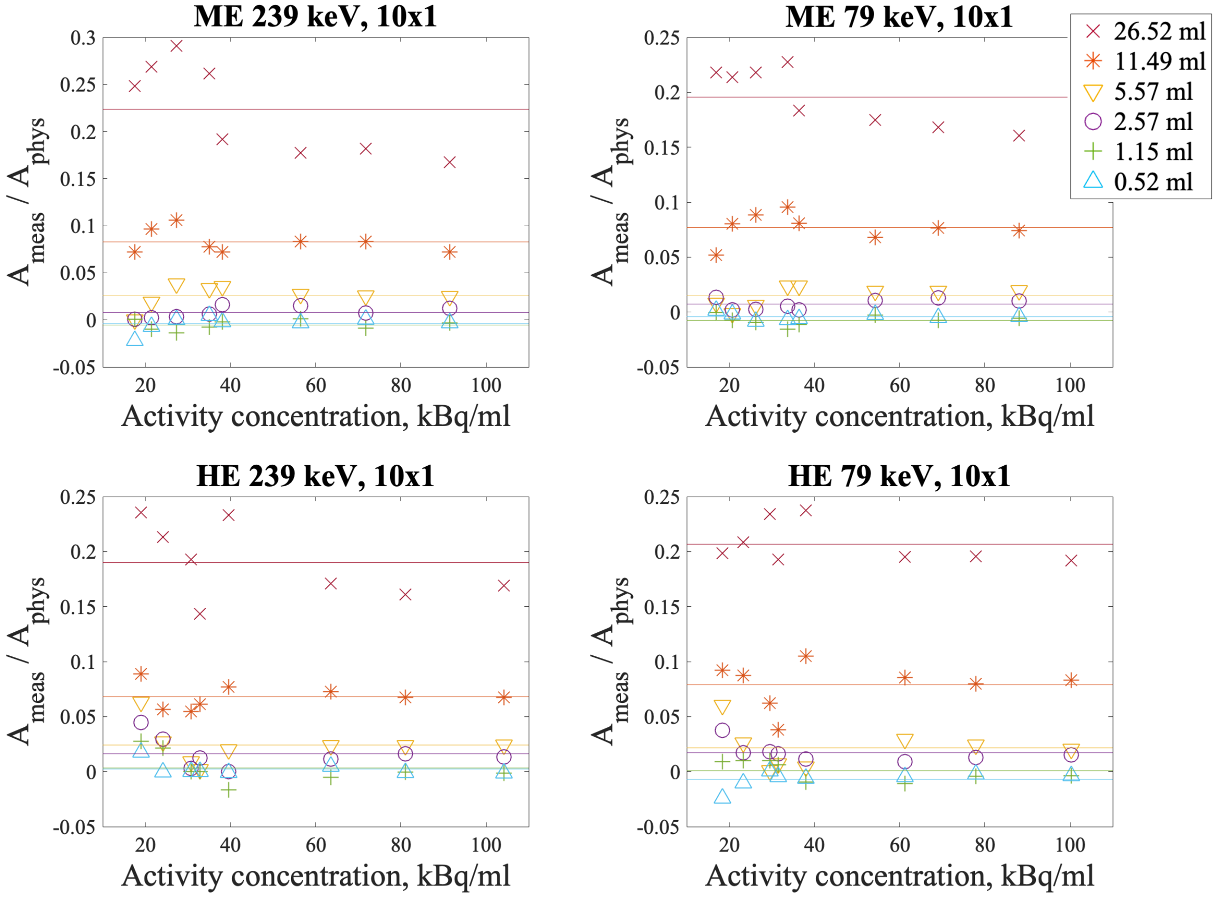

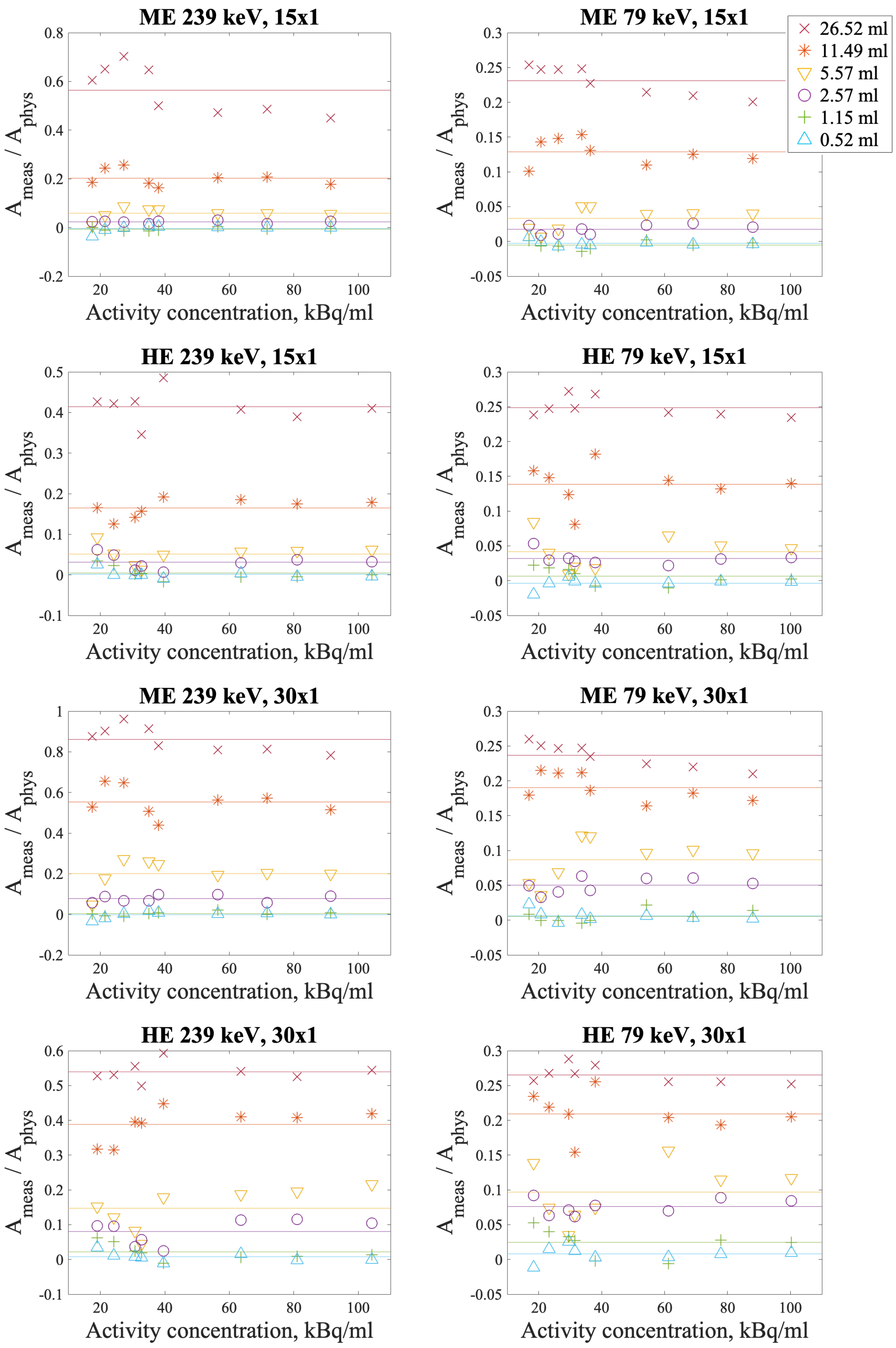
ME 239 keV, 15x1
ME 79 keV, 15x1
HE 239 keV, 15x1
HE 79 keV, 15x1
ME 239 keV, 30x1
ME 79 keV, 30x1
HE 239 keV, 30x1
HE 79 keV, 30x1
A_meas / A_phys
Activity concentration, kBq/ml
26.52 ml
11.49 ml
5.57 ml
2.57 ml
1.15 ml
0.52 ml

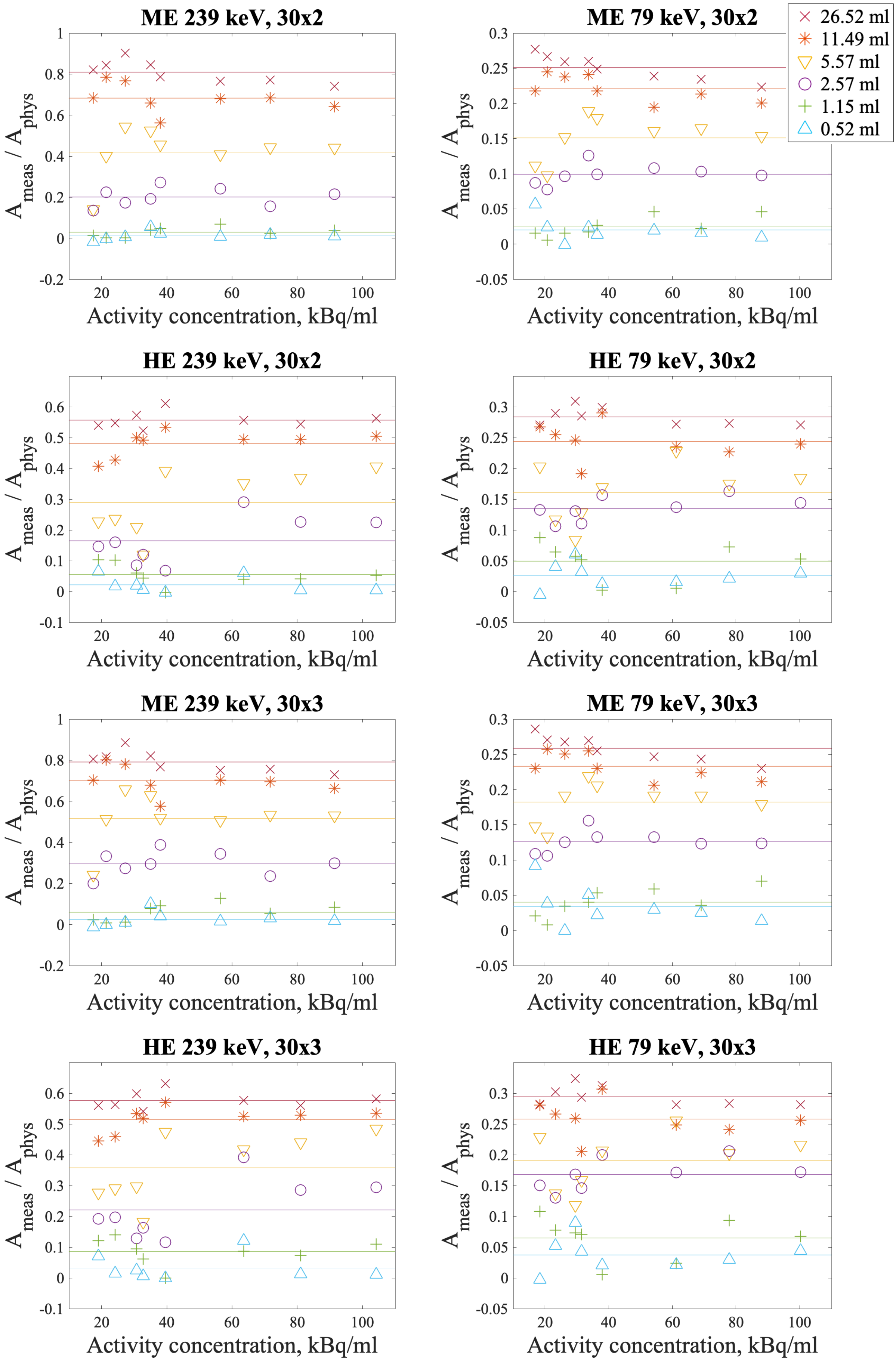
ME 239 keV, 30x2
ME 79 keV, 30x2
HE 239 keV, 30x2
HE 79 keV, 30x2
ME 239 keV, 30x3
ME 79 keV, 30x3
HE 239 keV, 30x3
HE 79 keV, 30x3
26.52 ml
11.49 ml
5.57 ml
2.57 ml
1.15 ml
0.52 ml
A_meas / A_phys
Activity concentration, kBq/ml

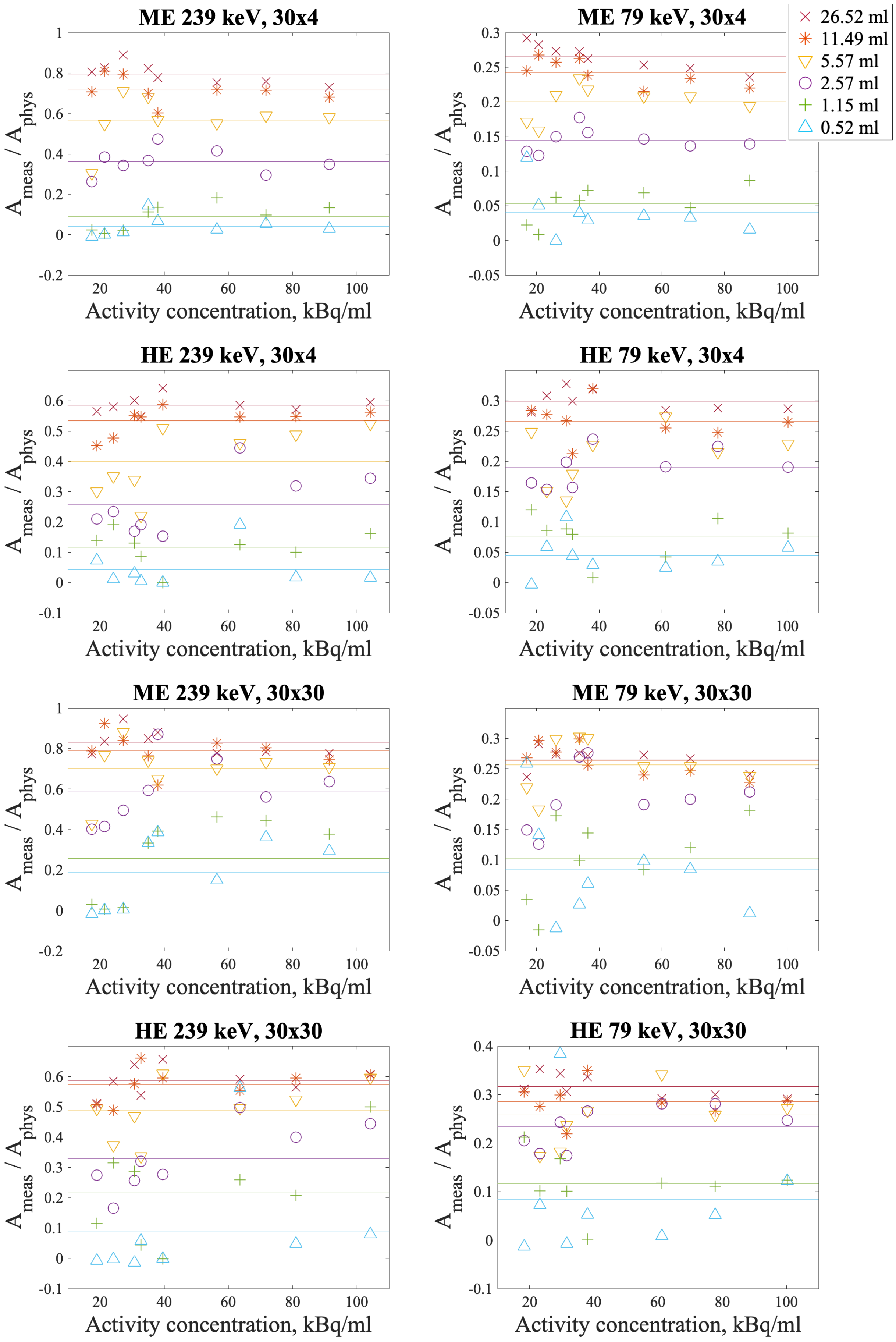
ME 239 keV, 30x4
ME 79 keV, 30x4
HE 239 keV, 30x4
HE 79 keV, 30x4
ME 239 keV, 30x30
ME 79 keV, 30x30
HE 239 keV, 30x30
HE 79 keV, 30x30
A_{meas} / A_{phys}
Activity concentration, kBq/ml
26.52 ml
11.49 ml
5.57 ml
2.57 ml
1.15 ml
0.52 ml

RC fits for all unfiltered reconstructions:

RC_{mean} plotted for the four imaging protocols for all the unfiltered reconstructions. Curve fits applied to RC_{mean} are also shown. Note that the y-axes differ. The fitting parameters and their associated uncertainties are presented in tables for each reconstruction.

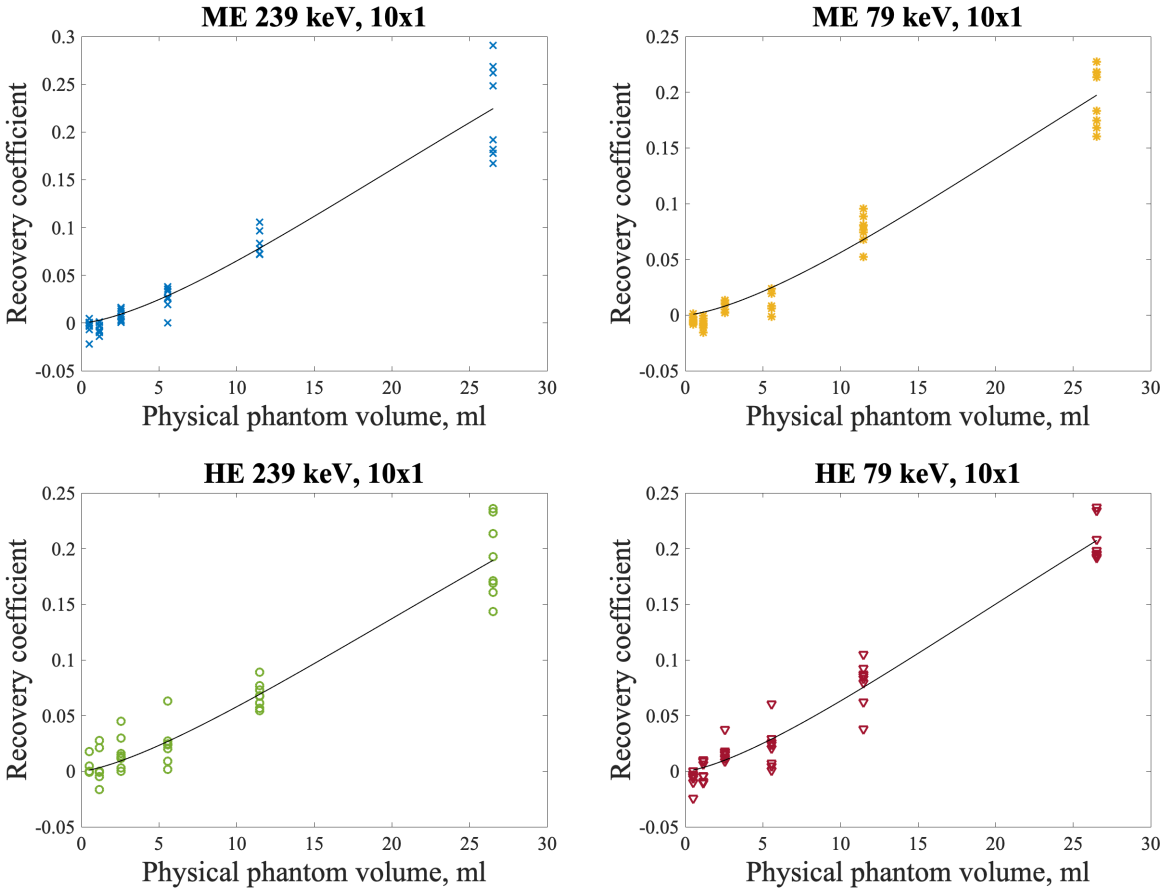

RC_{mean}, 10x1	Parameter	Value	Standard error	Fractional standard error
**ME 239 keV**	b_1	61.89 ml	4.86 ml	8%
	b_2	1.463	0.107	7%
**ME 79 keV**	b_1	69.48 ml	4.65 ml	7%
	b_2	1.456	0.083	6%
**HE 239 keV**	b_1	76.55 ml	7.18 ml	9%
	b_2	1.371	0.0997	7%
**HE 79 keV**	b_1	69.53 ml	4.72 ml	7%
	b_2	1.391	0.079	6%

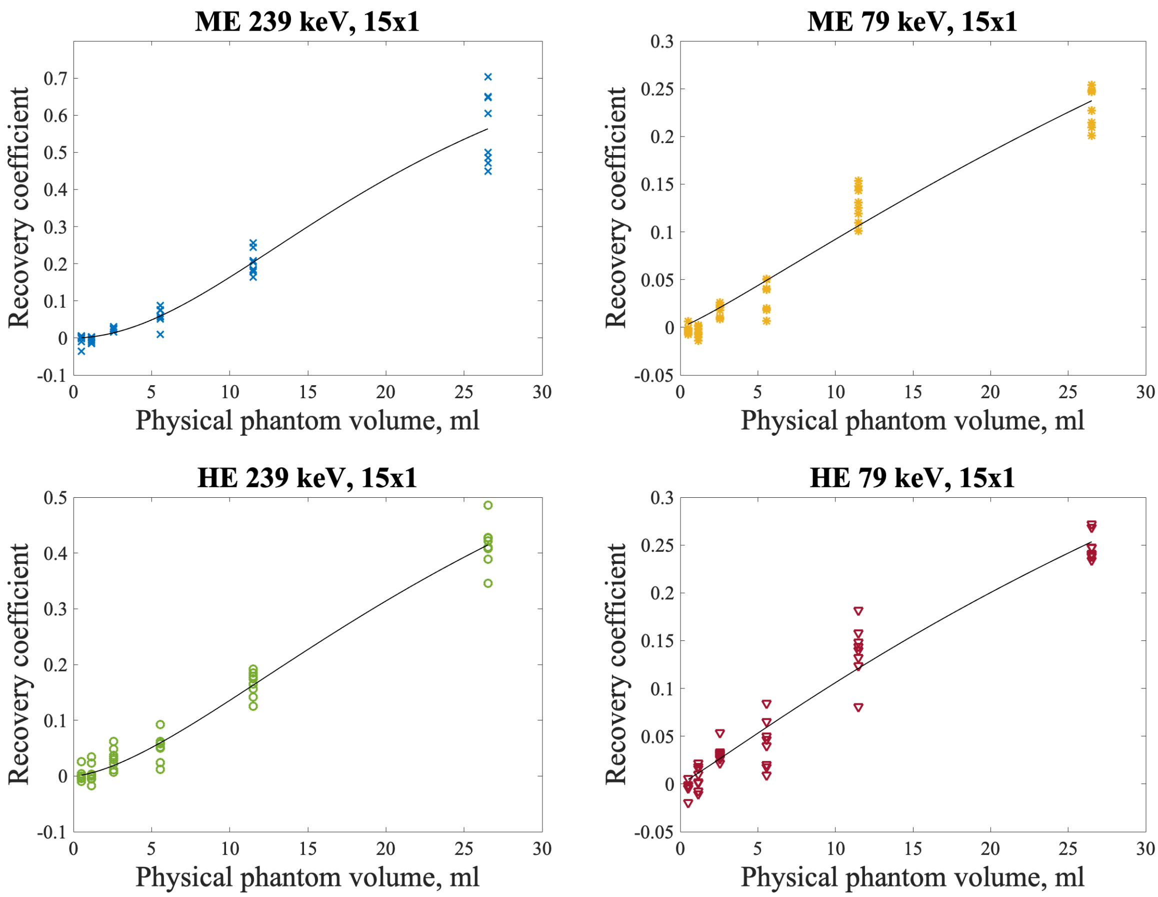

RC$_{mean}$, 15x1	**Parameter**	**Value**	**Standard error**	**Fractional standard error**
**ME 239 keV**	b_1	23.24 ml	0.66 ml	3%
	b_2	1.935	0.112	6%
**ME 79 keV**	b_1	73.27 ml	5.64 ml	8%
	b_2	1.148	0.064	6%
**HE 239 keV**	b_1	33.17 ml	0.94 ml	3%
	b_2	1.545	0.065	4%
**HE 79 keV**	b_1	72.24 ml	5.83 ml	8%
	b_2	1.078	0.0607	6%

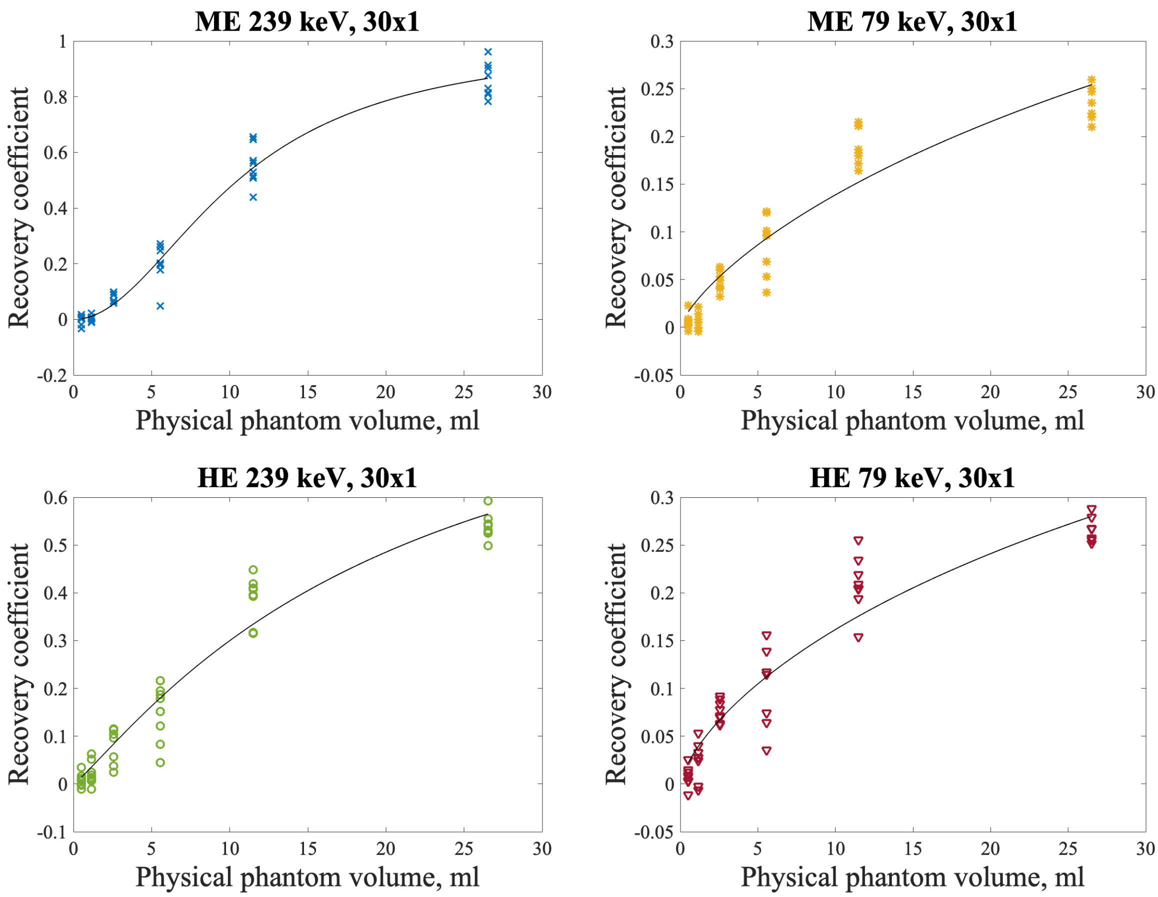

RC$_{mean}$, 30x1	**Parameter**	**Value**	**Standard error**	**Fractional standard error**
**ME 239 keV**	b_1	10.53 ml	0.29 ml	3%
	b_2	2.018	0.104	5%
**ME 79 keV**	b_1	107.7 ml	15.1 ml	14%
	b_2	0.768	0.051	7%
**HE 239 keV**	b_1	21.07 ml	0.99 ml	5%
	b_2	1.137	0.063	6%
**HE 79 keV**	b_1	98.28 ml	13.63 ml	14%
	b_2	0.7205	0.0475	7%

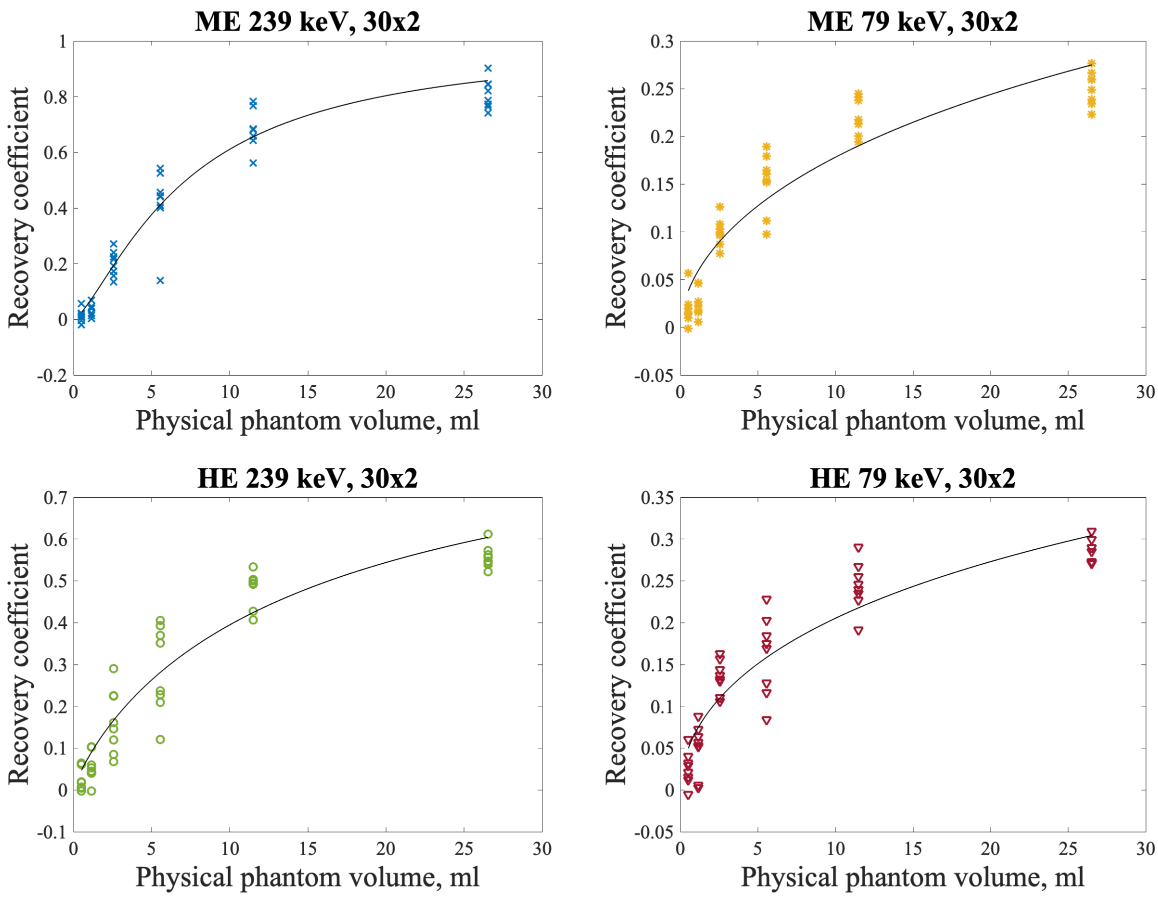

RC_{mean}, 30x2	**Parameter**	**Value**	**Standard error**	**Fractional standard error**
**ME 239 keV**	b_1	7.211 ml	0.339 ml	5%
	b_2	1.38	0.09	6%
**ME 79 keV**	b_1	144.1 ml	26.8 ml	19%
	b_2	0.5729	0.0397	7%
**HE 239 keV**	b_1	16.33 ml	1.23 ml	8%
	b_2	0.8709	0.0622	7%
**HE 79 keV**	b_1	122.7 ml	23.7 ml	19%
	b_2	0.5398	0.0395	7%

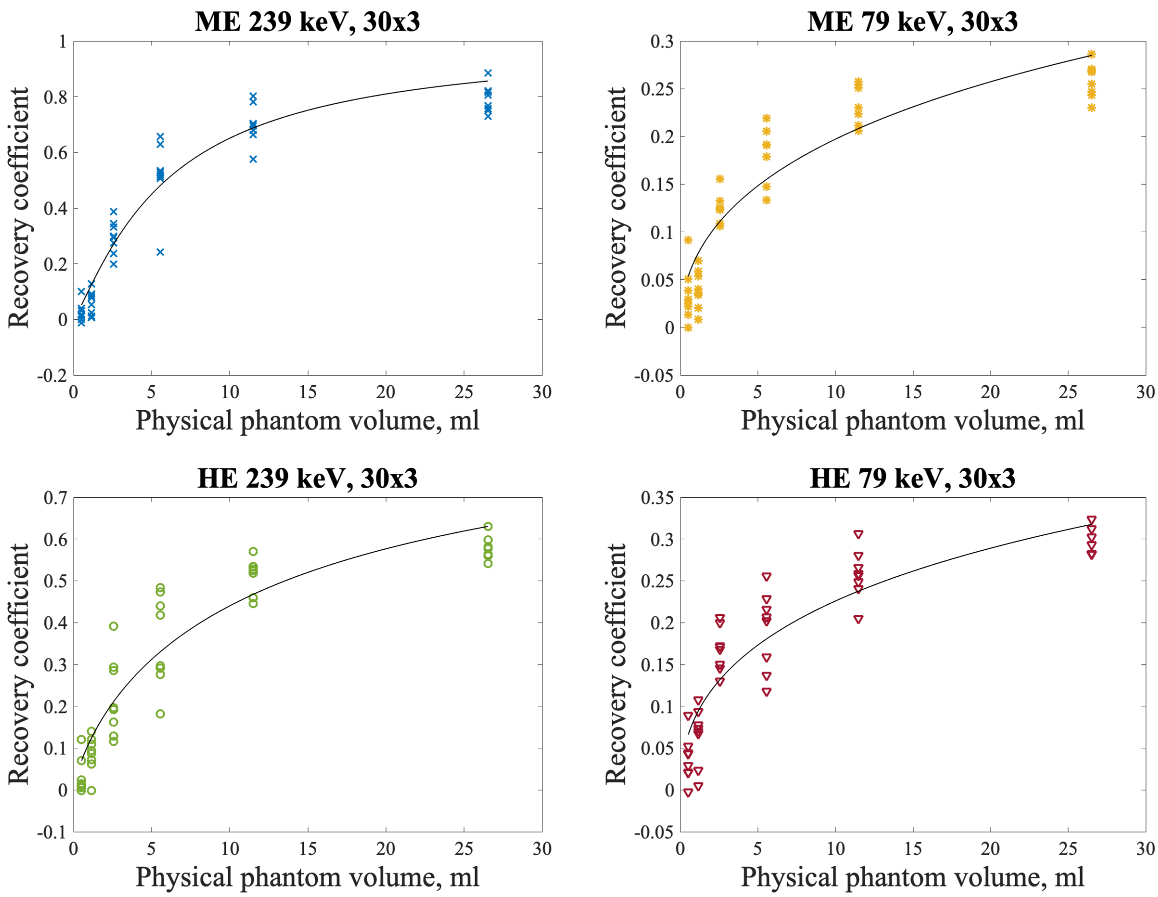

RC_{mean}, 30x3	**Parameter**	**Value**	**Standard error**	**Fractional standard error**
**ME 239 keV**	b_1	5.927 ml	0.346 ml	6%
	b_2	1.19	0.08	7%
**ME 79 keV**	b_1	169.3 ml	37.9 ml	22%
	b_2	0.4963	0.0371	7%
**HE 239 keV**	b_1	13.54 ml	1.148 ml	8%
	b_2	0.7932	0.0606	8%
**HE 79 keV**	b_1	131.1 ml	29.8 ml	23%
	b_2	0.4786	0.0383	8%

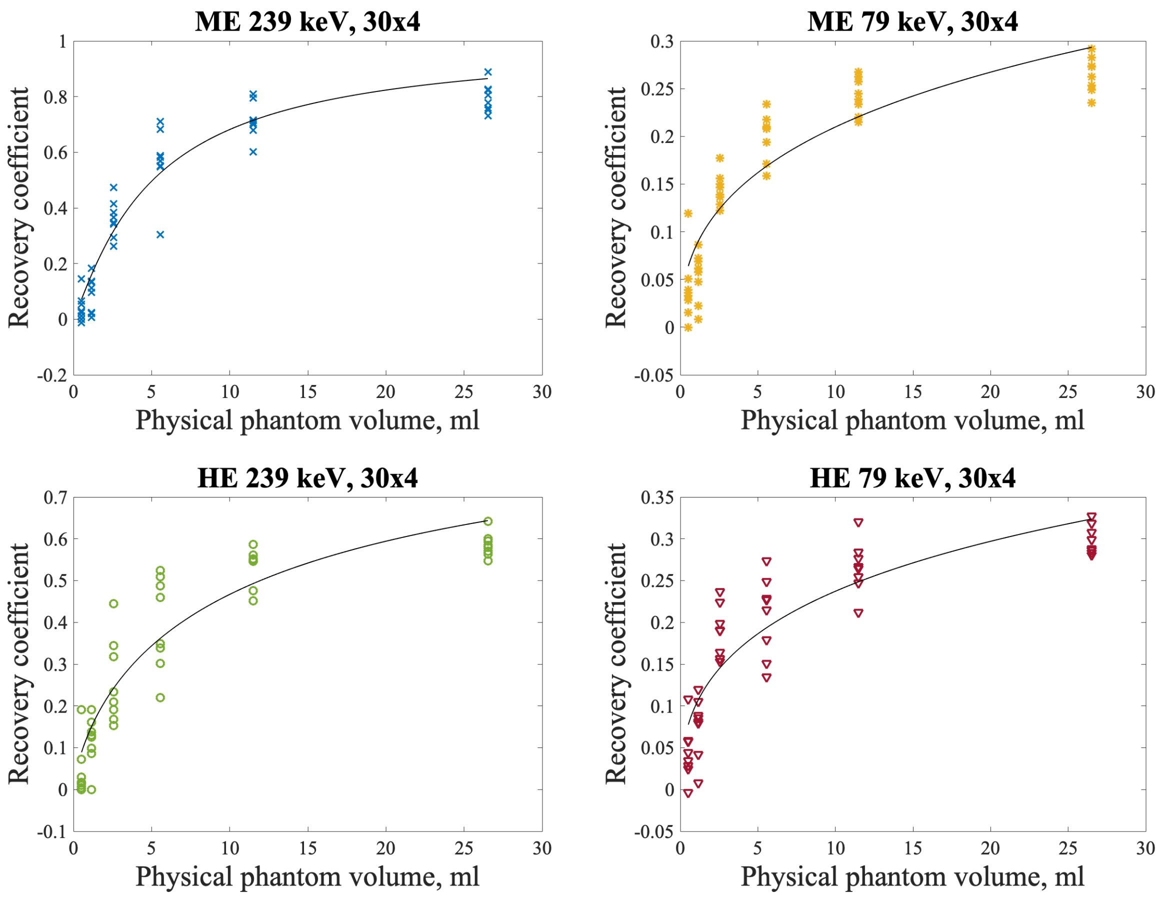

RC_{mean}, 30x4	**Parameter**	**Value**	**Standard error**	**Fractional standard error**
**ME 239 keV**	b_1	5.076 ml	0.329 ml	6%
	b_2	1.123	0.081	7%
**ME 79 keV**	b_1	180.6 ml	45.2 ml	25%
	b_2	0.4583	0.0365	8%
**HE 239 keV**	b_1	11.96 ml	1.13 ml	9%
	b_2	0.7414	0.0606	8%
**HE 79 keV**	b_1	140.6 ml	37.0 ml	26%
	b_2	0.4418	0.0388	9%

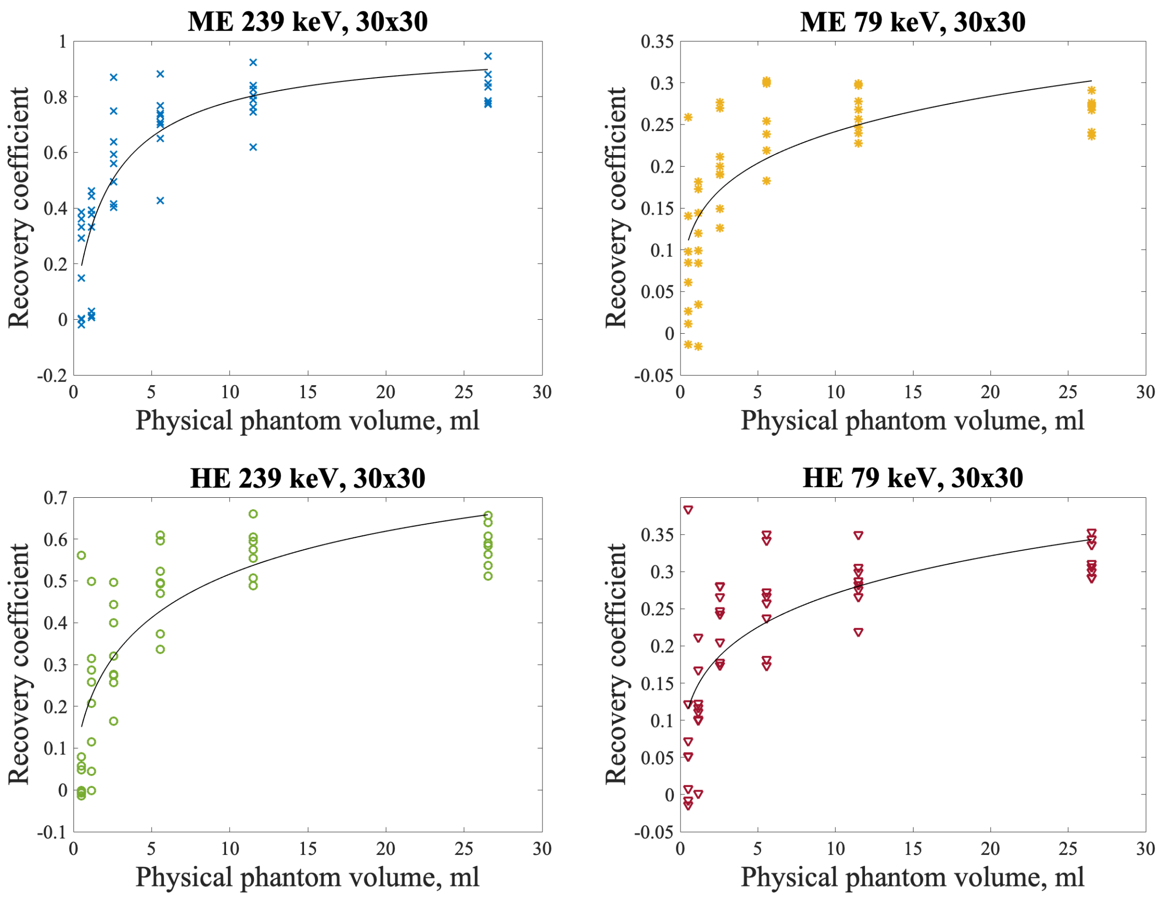

RC$_{mean}$, 30x30	Parameter	Value	Standard error	Fractional standard error
**ME 239 keV**	b_1	2.484 ml	0.317 ml	13%
	b_2	0.9165	0.1149	13%
**ME 79 keV**	b_1	374.8 ml	236.8 ml	63%
	b_2	0.3158	0.0479	15%
**HE 239 keV**	b_1	8.992 ml	1.438 ml	16%
	b_2	0.6062	0.0779	13%
**HE 79 keV**	b_1	168.3 ml	86.5 ml	51%
	b_2	0.3512	0.0532	15%

RC fits for all unfiltered reconstructions:

RC_{mean} and RC_{max} plotted for the four imaging protocols for all the unfiltered reconstructions. Curve fits applied to RC_{mean} are also plotted. Note that the y-axes differ.

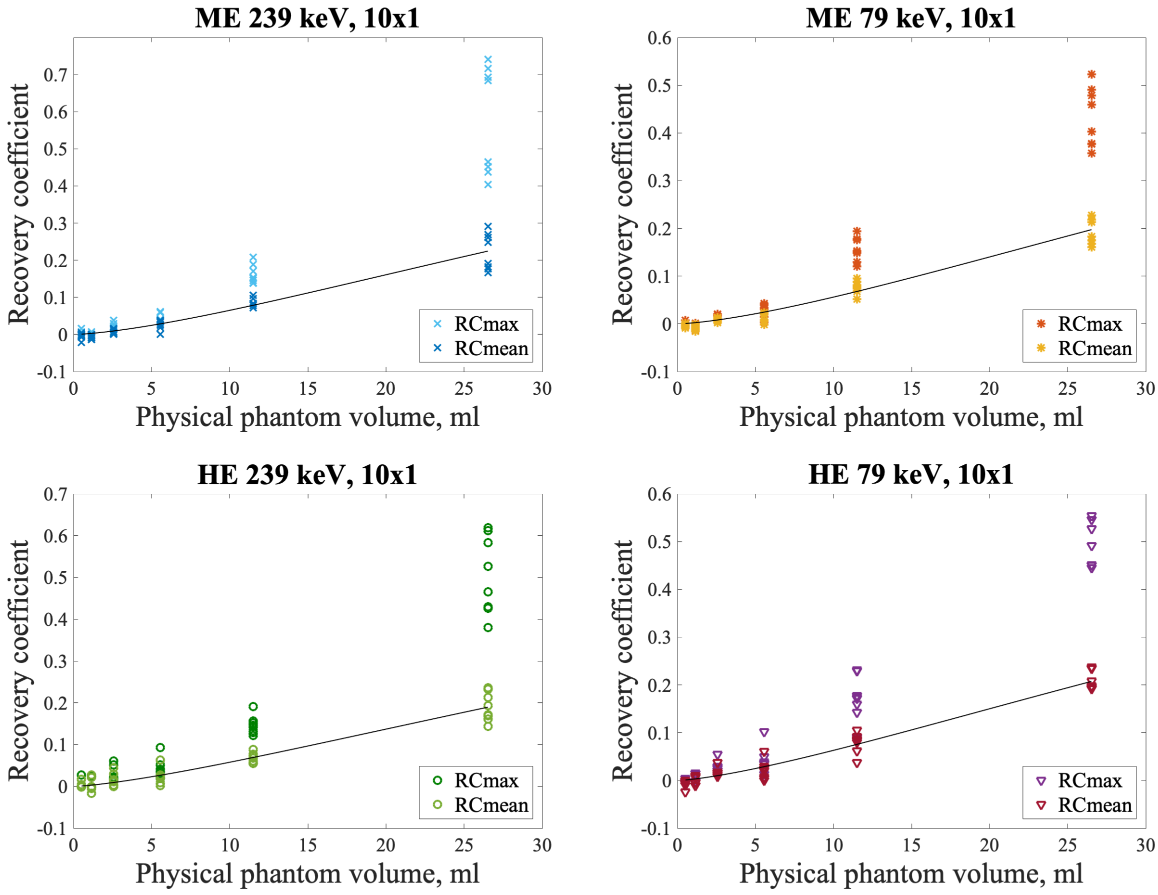

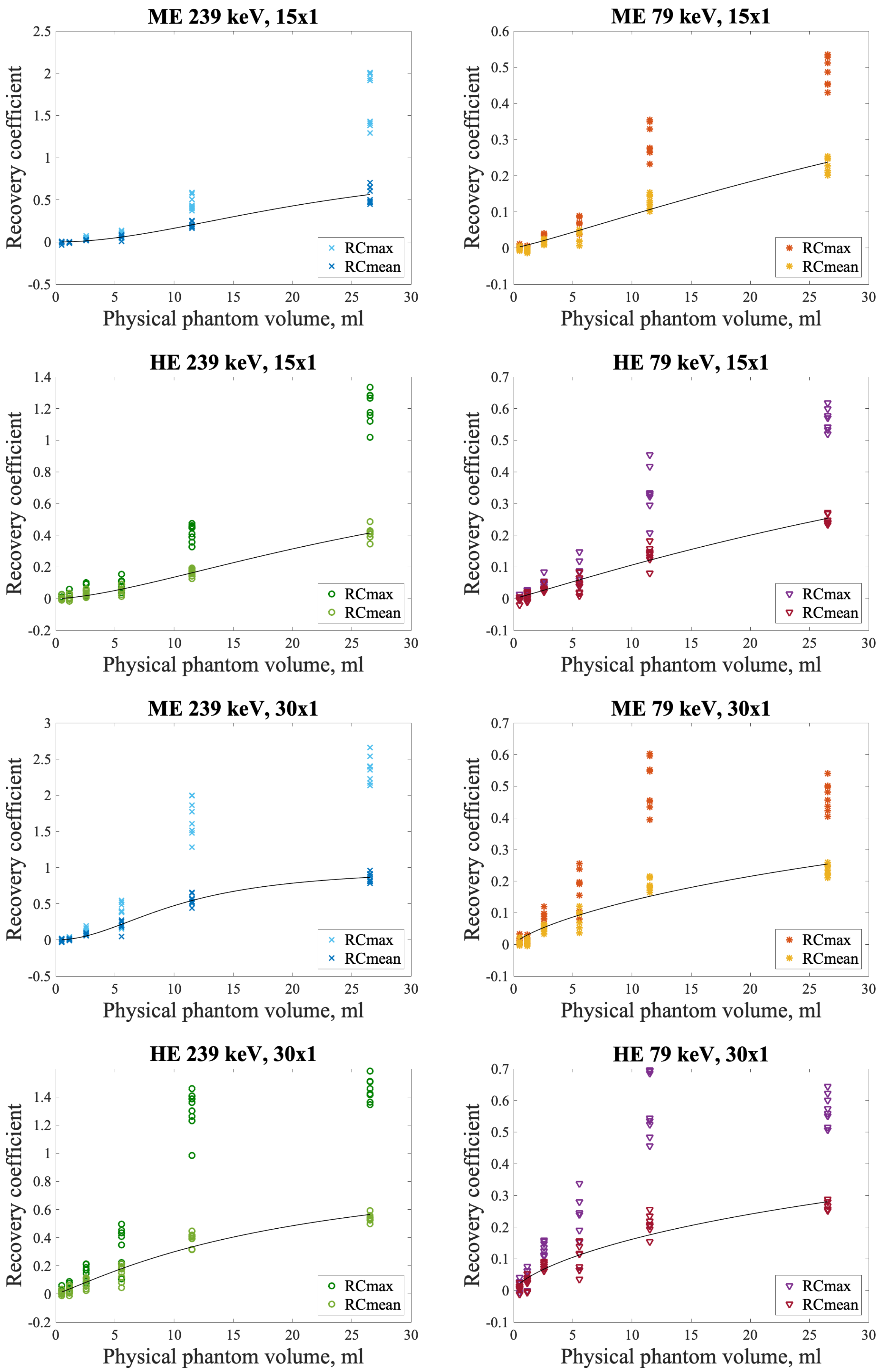
ME 239 keV, 15x1
ME 79 keV, 15x1
HE 239 keV, 15x1
HE 79 keV, 15x1
ME 239 keV, 30x1
ME 79 keV, 30x1
HE 239 keV, 30x1
HE 79 keV, 30x1
Recovery coefficient
Physical phantom volume, ml
RCmax
RCmean

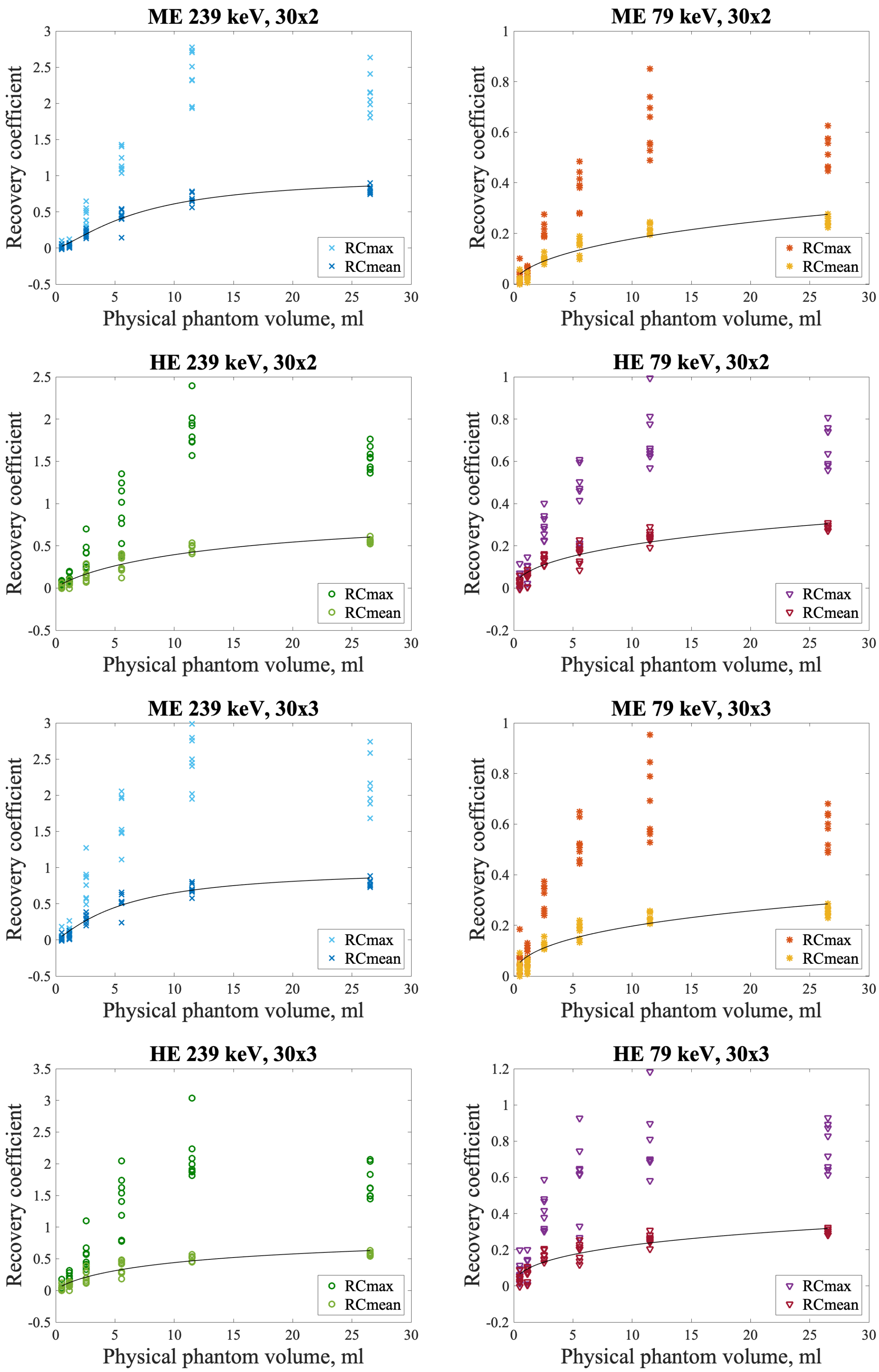
ME 239 keV, 30x2
ME 79 keV, 30x2
HE 239 keV, 30x2
HE 79 keV, 30x2
ME 239 keV, 30x3
ME 79 keV, 30x3
HE 239 keV, 30x3
HE 79 keV, 30x3
Recovery coefficient
Physical phantom volume, ml
RCmax
RCmean

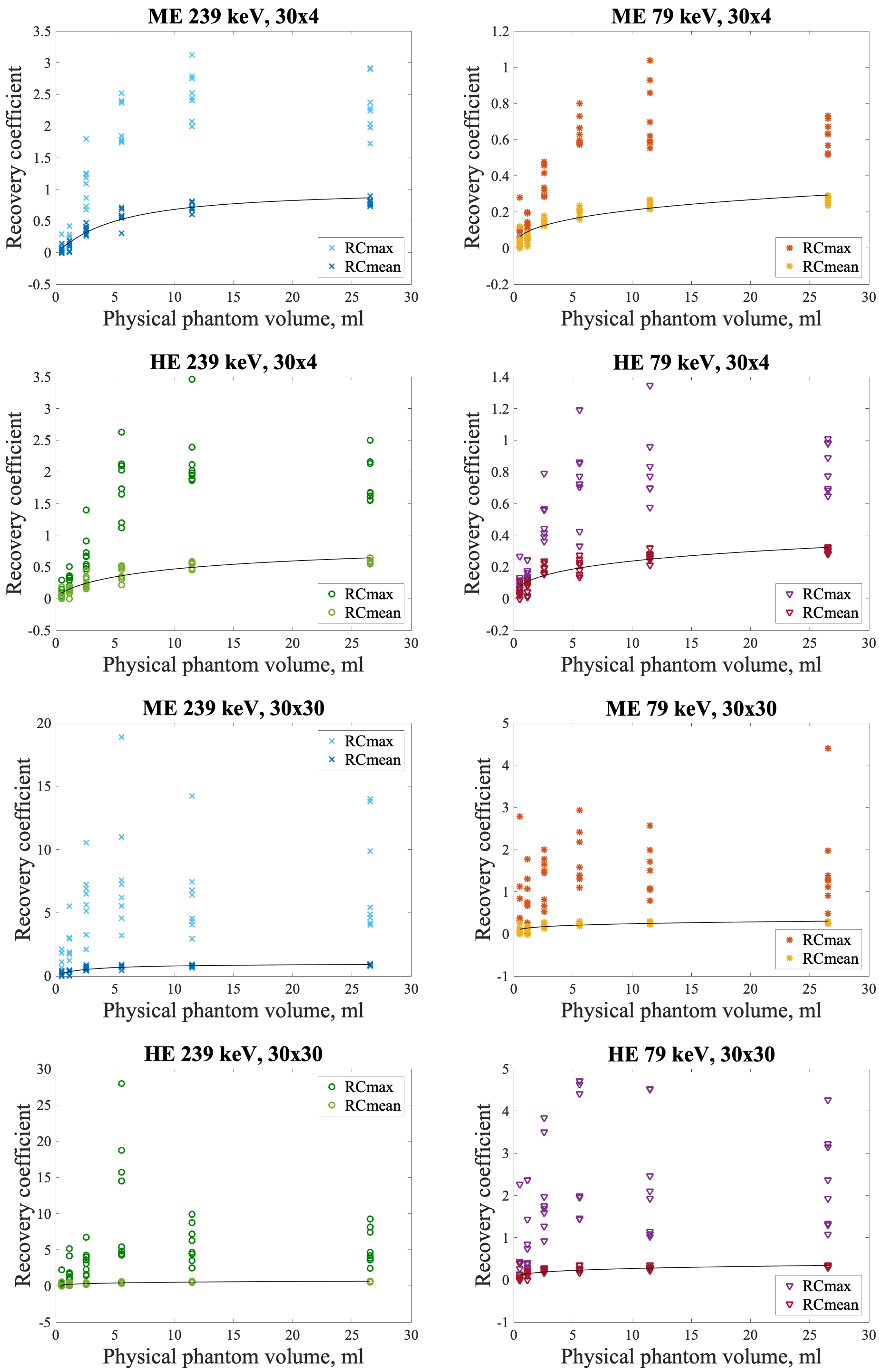
ME 239 keV, 30x4
Recovery coefficient
Physical phantom volume, ml
RCmax
RCmean
ME 79 keV, 30x4
Recovery coefficient
Physical phantom volume, ml
RCmax
RCmean
HE 239 keV, 30x4
Recovery coefficient
Physical phantom volume, ml
RCmax
RCmean
HE 79 keV, 30x4
Recovery coefficient
Physical phantom volume, ml
RCmax
RCmean
ME 239 keV, 30x30
Recovery coefficient
Physical phantom volume, ml
RCmax
RCmean
ME 79 keV, 30x30
Recovery coefficient
Physical phantom volume, ml
RCmax
RCmean
HE 239 keV, 30x30
Recovery coefficient
Physical phantom volume, ml
RCmax
RCmean
HE 79 keV, 30x30
Recovery coefficient
Physical phantom volume, ml
RCmax
RCmean

Fractional uncertainties on RCs for unfiltered images:

Fractional uncertainties on RC_{mean} (left) and RC_{max} (right) plotted for the four imaging protocols against reconstruction updates for the six spheres on unfiltered images.

26.52 ml volume:

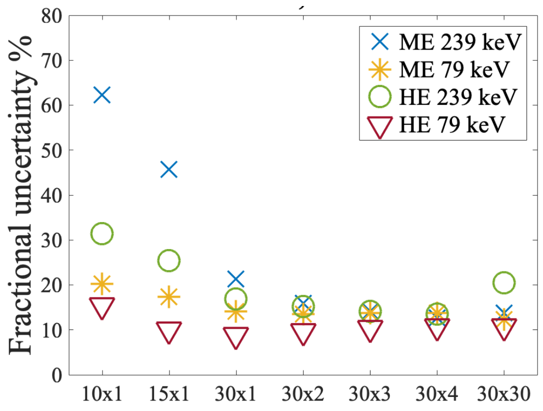

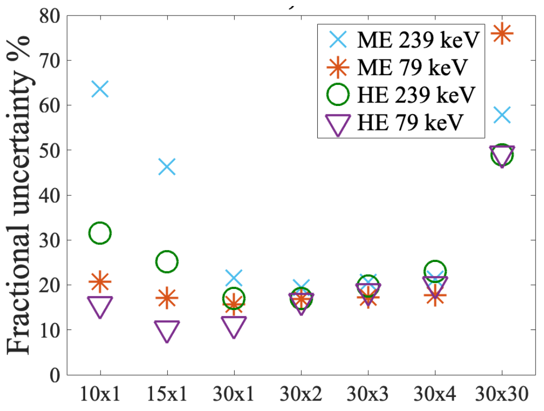

11.49 ml volume:

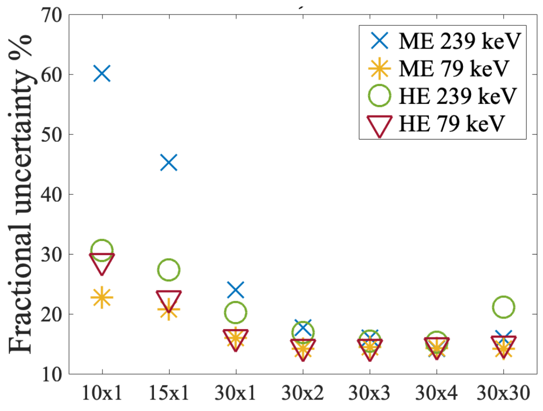

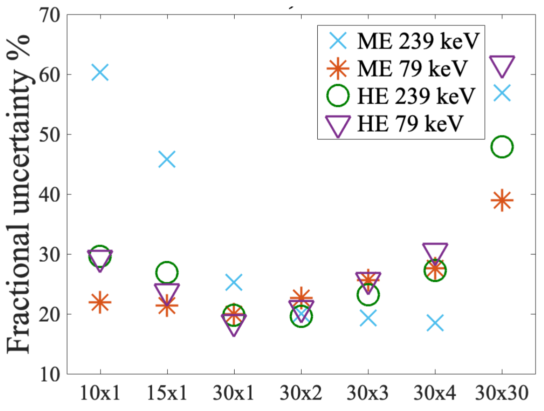

5.57 ml volume:

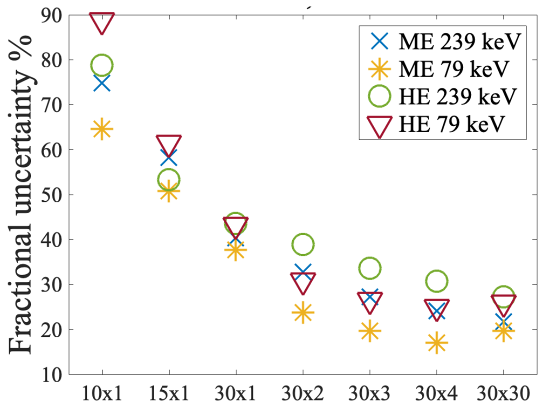

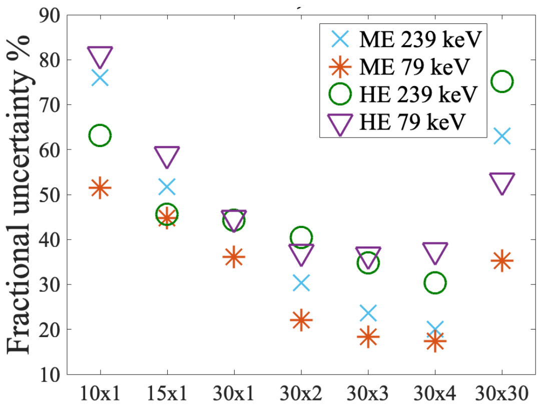

2.57 ml volume:

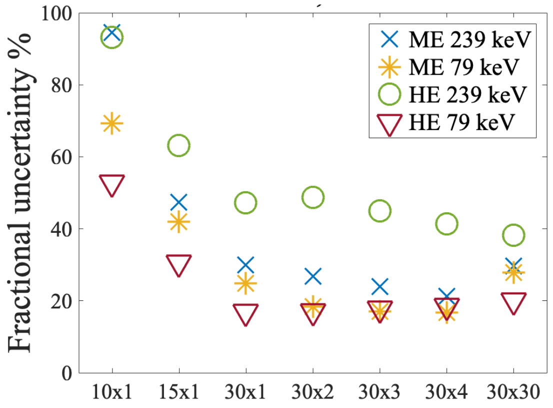

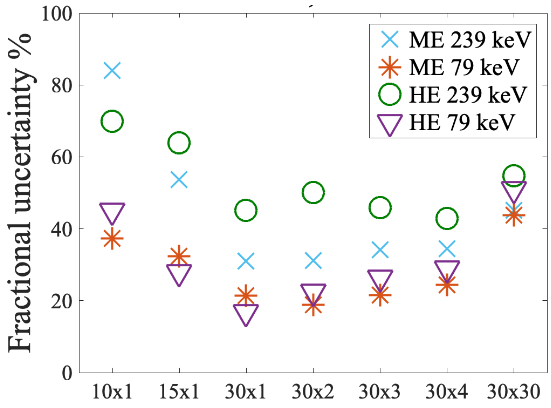

1.15 ml volume:

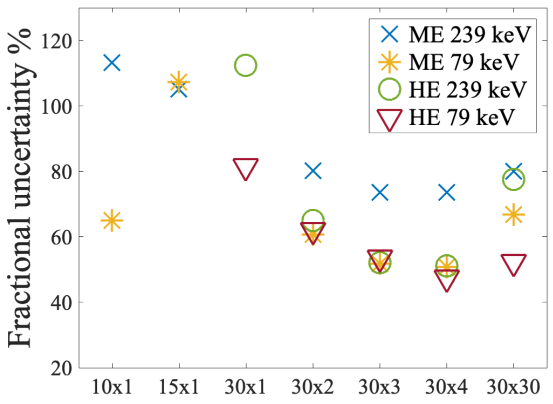

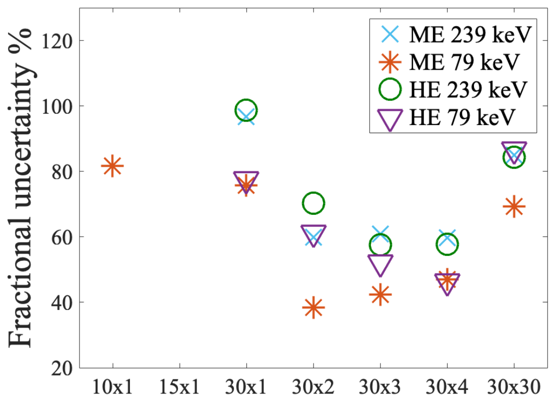

0.52 ml volume:

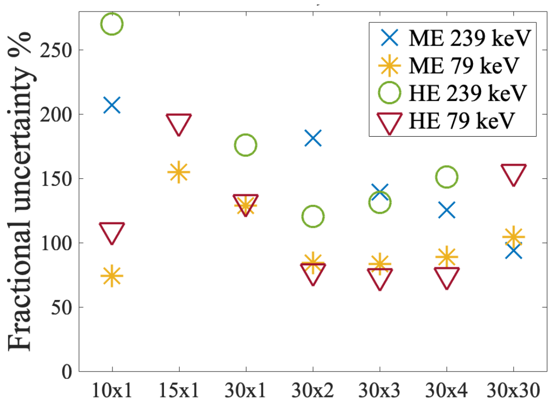

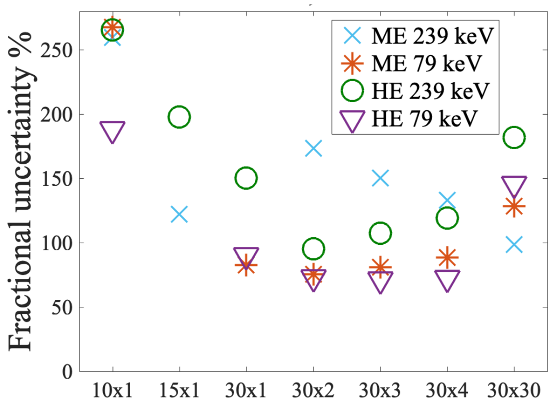

Examples of unfiltered images for the four imaging protocols with a high activity concentration in the spheres:

Unfiltered images for the 15x1, 30x4, and 30x30 reconstruction shown for the four imaging protocols with approximately 100 kBq/ml Pb-212 in the spheres.

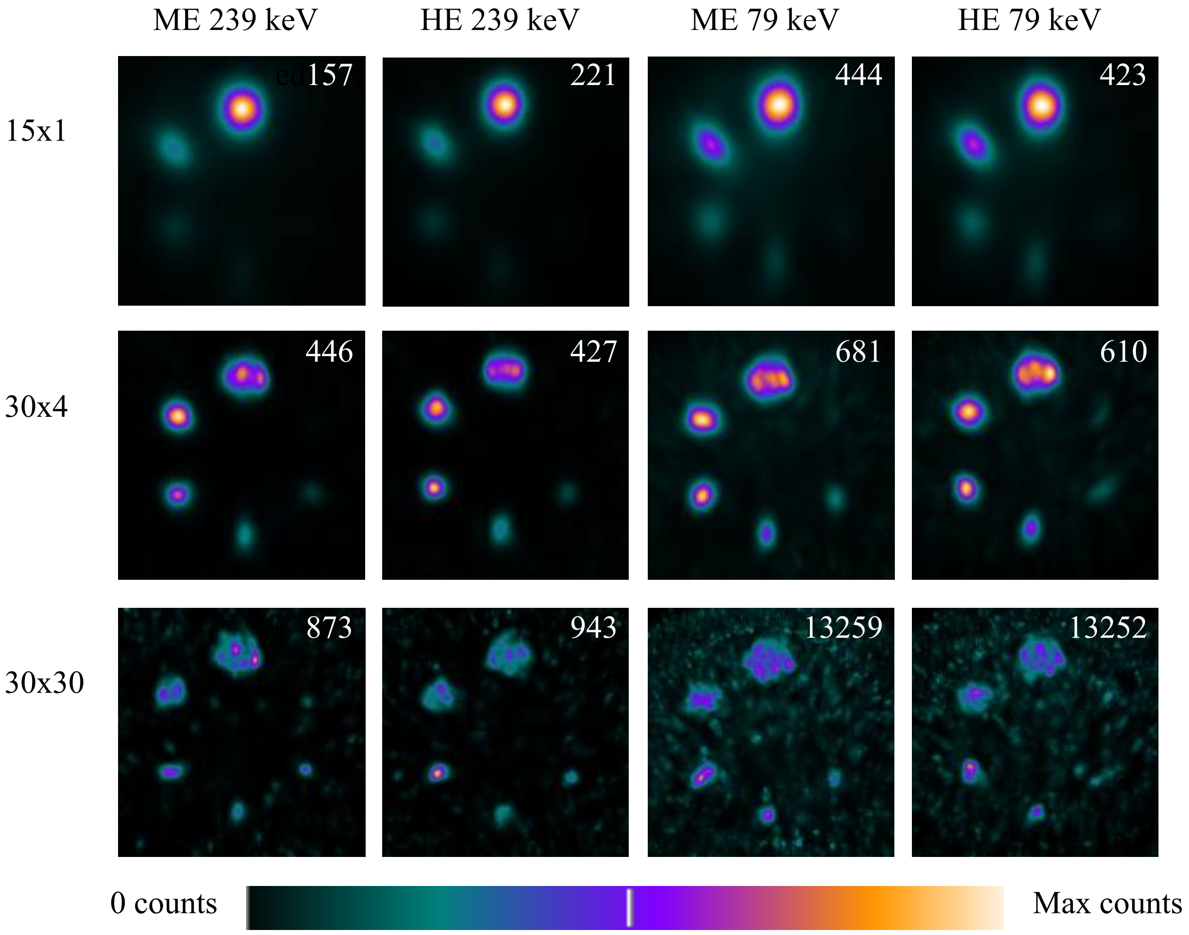

Examples of unfiltered images for the four imaging protocols with a low activity concentration in the spheres:

Unfiltered images for the 15x1, 30x4, and 30x30 reconstruction shown for the four imaging protocols with approximately 17 kBq/ml Pb-212 in the spheres.

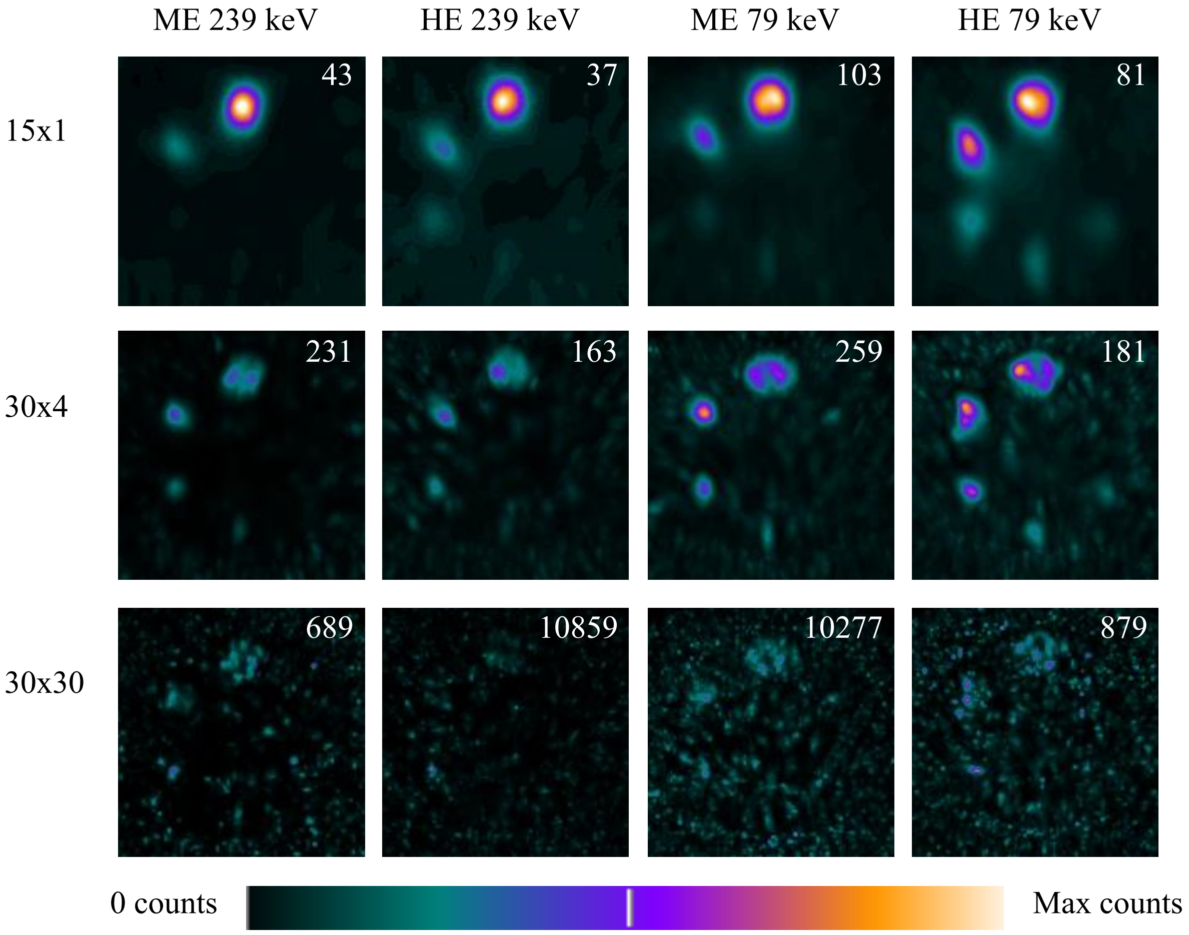

Contrast for the different imaging protocols for all the unfiltered reconstructions:

Contrast plotted against sphere diameter plotted for the different unfiltered reconstructions. Each of the imaging protocols have a panel.

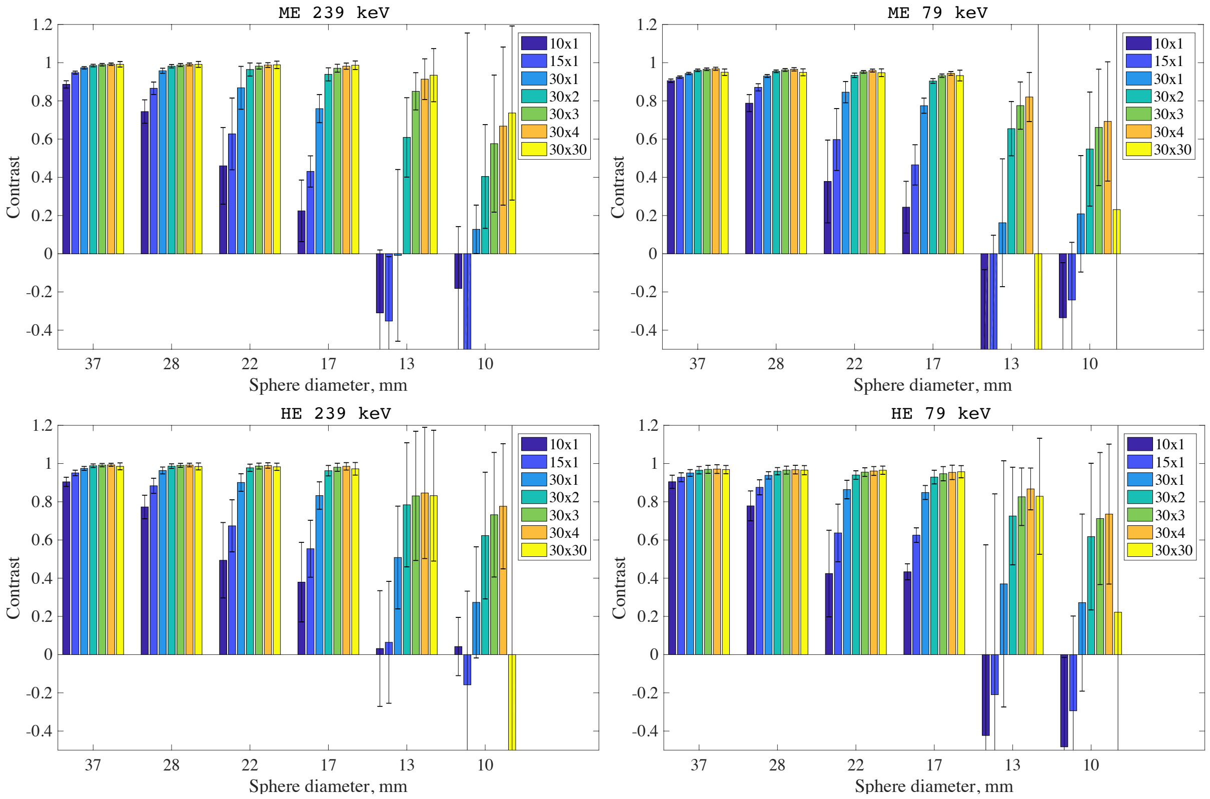

Supplement: Supplementary file 1 — Additional file 1: All supplementary materials with results from reconstructions not shown in the main paper are found in Additional file 1. Pages 1–12 include all materials from the reconstructions with a 12-mm Gaussian filter applied which were not included in the paper. Pages 13–34 include all materials from the reconstructions without filters applied. Calibration factors for all reconstructions are given in the tables on pages 1 (filtered) and 13 (unfiltered). The equivalent plots as that shown in Fig. 2 c are shown for the other reconstructions on page 2 (filtered) and pages 14–15 (unfiltered). The unfiltered versions of Fig. 3 a and b are found on page 13. Versions of Fig. 4 for other reconstructions are shown on pages 3–5 (filtered) and 15–18 (unfiltered). Recovery plots as shown in Fig. 5 and tables equivalent to Table 1 for other reconstructions are given on pages 6–11 (filtered) and 19–29 (unfiltered). The equivalent plots as those given in Fig. 6 without filters are given on pages 30–31 and the filtered version for the smallest sphere is given on page 12. The unfiltered versions of the images in Fig. 7 and Fig. 8 are shown on page 32 and 33, respectively. The version of Fig. 9 without a filter is given on page 34. [file 40658_2022_481_MOESM1_ESM.pdf]
